# Quantification of Kuramoto Coupling Between Intrinsic Brain Networks Applied to fMRI Data in Major Depressive Disorder

**DOI:** 10.3389/fncom.2022.729556

**Published:** 2022-03-03

**Authors:** Lena G. Bauer, Fabian Hirsch, Corey Jones, Matthew Hollander, Philipp Grohs, Amit Anand, Claudia Plant, Afra Wohlschläger

**Affiliations:** ^1^Research Network Data Science, University of Vienna, Vienna, Austria; ^2^Departement of Neuroradiology, Klinikum Rechts der Isar, Technical University of Munich, Munich, Germany; ^3^TUMNIC, Klinikum Rechts der Isar, Technical University of Munich, Munich, Germany; ^4^Faculty of Mathematics, University of Vienna, Vienna, Austria; ^5^Center for Behavioral Health, Cleveland Clinic, Cleveland, OH, United States; ^6^Faculty of Computer Science, University of Vienna, Vienna, Austria

**Keywords:** Kuramoto model, functional connectivity, synchronization, fMRI, major depressive disorder (MDD)

## Abstract

Organized patterns of system-wide neural activity adapt fluently within the brain to adjust behavioral performance to environmental demands. In major depressive disorder (MD), markedly different co-activation patterns across the brain emerge from a rather similar structural substrate. Despite the application of advanced methods to describe the functional architecture, e.g., between intrinsic brain networks (IBNs), the underlying mechanisms mediating these differences remain elusive. Here we propose a novel complementary approach for quantifying the functional relations between IBNs based on the Kuramoto model. We directly estimate the Kuramoto coupling parameters (*K*) from IBN time courses derived from empirical fMRI data in 24 MD patients and 24 healthy controls. We find a large pattern with a significant number of *K*s depending on the disease severity score Hamilton D, as assessed by permutation testing. We successfully reproduced the dependency in an independent test data set of 44 MD patients and 37 healthy controls. Comparing the results to functional connectivity from partial correlations (*FC*), to phase synchrony (*PS*) as well as to first order auto-regressive measures (*AR*) between the same IBNs did not show similar correlations. In subsequent validation experiments with artificial data we find that a ground truth of parametric dependencies on artificial regressors can be recovered. The results indicate that the calculation of *K*s can be a useful addition to standard methods of quantifying the brain's functional architecture.

## 1. Introduction

The human brain is a complex adaptive system in which a stable neuronal substrate of gray and white matter architecture allows for a vast array of cognitive sets. At any moment integrative overall network interaction defines attainable cognitive sets as well as the degree of flexibility to react to outer stimuli (Sporns et al., 2004; Deco et al., 2008; Breakspear, 2017). Empirically, on the one hand a structural connectome can be described (Sporns et al., 2005), and complementary to that functional imaging allows for assessing the functional architecture which is in parts defined by processes of chemical connectivity depending on the status of the various transmitter systems of the brain (Shine et al., 2019). Different measures have been proposed to quantify the complex interplay of brain areas measured with functional magnetic resonance imaging (fMRI) including statistical measures of coherence (functional connectivity; Friston, 2011), phase coherence (Glerean et al., 2012; Deco and Kringelbach, 2016; Cabral et al., 2017), and models of first order auto-regressive representation (Liégeois et al., 2017, 2019). Still a full understanding of how a brain state arises from neuronal underpinnings of structural and chemical connectivity remains elusive. Alternative approaches might help to fill into this gap. Table 1 contains all abbreviations used in this paper.

**Table 1:** Abbreviations.

**Section**	**Abbreviation**	**Meaning**
Connectivity	*K*	Kuramoto coupling parameters
Measures	*FC*	Functional connectivity from partial correlations
	(*I*)*PS*	(Instantaneous) phase synchrony
	*AR*	First order auto-regressive measures
Neuroscience	fMRI	Functional magnetic resonance imaging
	MD	Major depressive disorder
	MDE	Major depressive episode
	HC	Healthy controls
	IBN	Intrinsic brain network
	BG	Basal ganglia network
	Ham-D	Hamilton Rating Scale for Depression
Methods	ICA	Independent component analysis
	KM	Kuramoto model
	ODE	Ordinary differential equation
	LES	Linear equation system
Simulation	IC	Inside correlation pattern coefficients
	BC	Bridging coefficients
	RC	Reference coefficients

The Kuramoto model of coupled oscillators (Okuda and Kuramoto, 1991) has been introduced to neuroscience as one potential generative model governing fluctuating oscillations in large-scale cortical circuits (Breakspear et al., 2010; Cocchi et al., 2016). The model poses that the differences in time course phases between any two oscillators are causal to phase readjustments at both ends (Okuda and Kuramoto, 1991).

**Figure 1:**
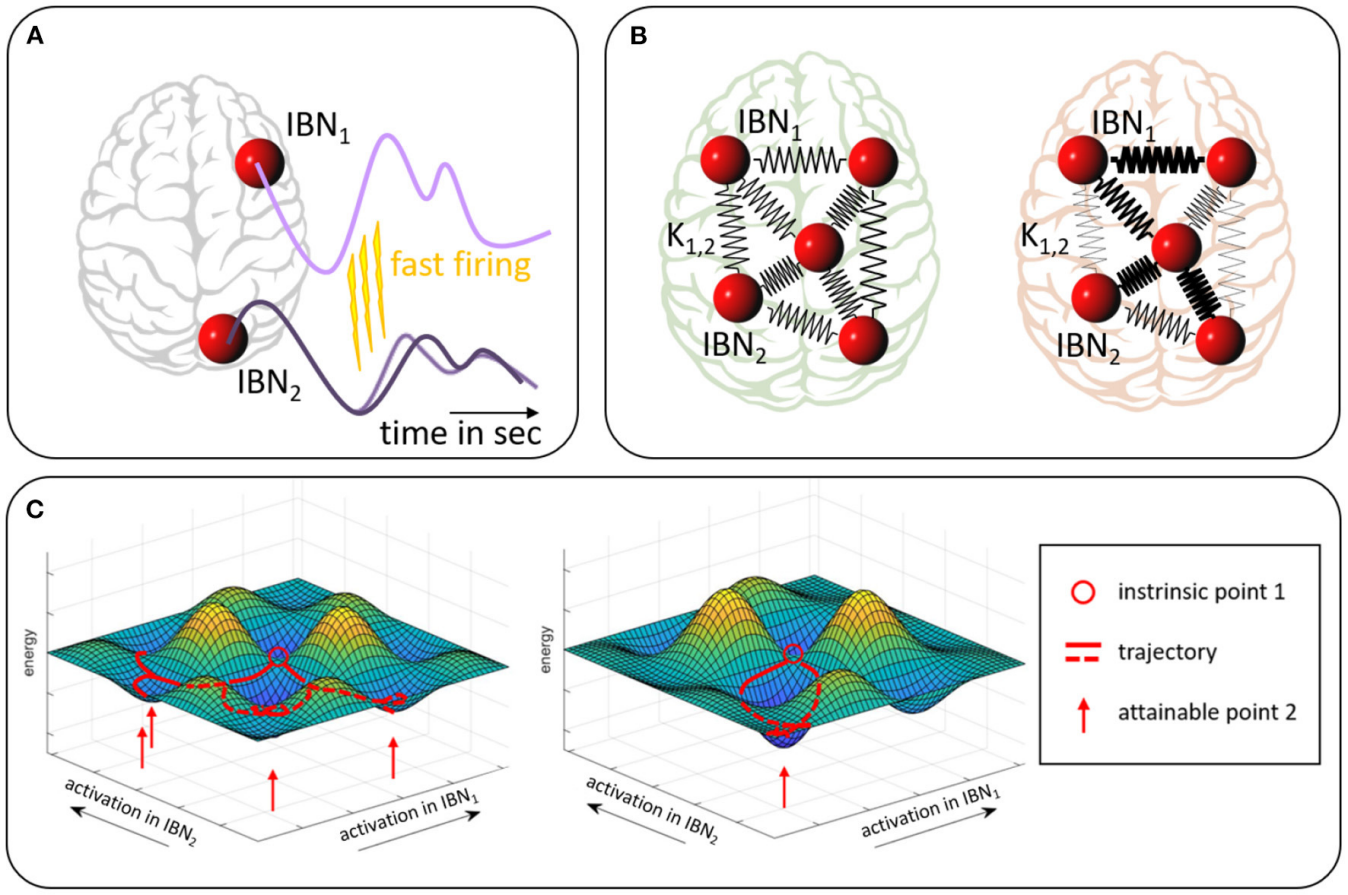
Sketch of the model assumption. **(A)** Excitatory neuronal firing from IBN_1_ on a fast time scale leads to an earlier signal rise in IBN_2_ which effectively means a phase readjustment in the slow BOLD time course of the targeted IBN. Conversely, inhibitory firing would lead to a phase adjustment in the form of phase repulsion. **(B)** Pair-wise Kuramoto phase couplings of IBNs, symbolized by spring constants (here undirected), determine network-wide dynamics and are altered in pathology (compare red vs. green). **(C)** Energy landscape on a very reduced subspace of only two IBNs. Different activation constellations of the two IBNs (x- and y-axis) are associated with different energy levels (z-axis). Intrinsic information flow between the IBNs favors selective co-activations and penalizes others. The red circle indicates an arbitrary intrinsic state (i.e., co-activation constellation between IBNs), red lines indicate trajectories from this state which are favored by the landscape, red arrows indicate a set of states likely to be attained under the prevailing structural and synaptic conditions. The landscape is based on the underlying neuronal and synaptic connectivity. Minor adjustments to an overall stable energy landscape (compare left and right) may impact on fast firing intensity and thereby on Kuramoto coupling parameters *K*_*i,j*_. This might allow for or subdue more versatile co-activation patterns. Widely projecting transmitter systems bear the potential of widespread moderate adjustments to the energy landscape.

While classical functional connectivity analyses look for first order statistical associations, the application of the Kuramoto model to fMRI data employs a more specific, yet simple, biophysiological model, i.e., it addresses the issue of a slow BOLD response to fast neuronal processes. As depicted in Figure 1A an event of fast neuronal firing in one region would cause an attraction of the phase of the fMRI signal in a second region receiving excitatory neuronal projections from the first one. Conversely, repetitive inhibitory impact from one region onto the other on a fast time scale would cause phase repulsion on the fMRI time scale. In this broader conceptualization, the Kuramoto coupling strengths can serve as empirical measures even without the assumption that the brain regions are perfect oscillators.

The overall energy landscape, which determines the likelihood of any pattern of simultaneously active brain regions (Okuda and Kuramoto, 1991; Shine, 2020), depends on the individual coupling strengths (see Figure 1B). Minor, but widespread modifications in the coupling strengths result in changes of this landscape and thereby in a notably different spectrum of co-activations. These ultimately have to be understood as different brain processes, i.e., alterations in thought and behavior. The idea is illustrated in a conceptual sketch in Figure 1C. In this view Kuramoto coupling between brain areas could for instance be changed by underlying changes in the transmitter system status which impacts on the amount of fast firing. The model, therefore, might offer a distinct complementary approach to other existing ones based on an appealing generative model.

Major depressive disorder (MD) is associated with experiences of depressed mood, with impaired cognition, energy loss, vegetative symptoms, and suicidal thoughts (American Psychiatric Association, 2013). This spectrum of diverse symptoms suggests a likely involvement of several distinct neural circuits in creating an aberrant brain state (Northoff, 2016). MD is not associated with a major focal brain lesion, but is frequently associated with alterations in synaptic, chemical rather than structural connectivity, in particular with monoamine dysfunction, which has been investigated in detail in animal and human models of depression (Cooper et al., 1991; Delgado et al., 1994; Hamon and Blier, 2013). Most antidepressants act on monoamine re-uptake mechanisms or monoamine post-synaptic receptors (Anand and Charney, 1997). Monoamine transmitter systems are mainly centrally controlled by brainstem nuclei, which exert wide spread influence via broad projections to nearly all cortico-limbic regions (Goldman-Rakic et al., 1989; Robbins and Arnsten, 2009; Jacob and Nienborg, 2018). Although they may be central to the generative mechanisms determining pathological alterations of the brain's energy landscape their direct impact on fMRI measures is difficult to establish. Therefore, a method investigating moderate but wide spread changes in the brain's functional architecture focussing on causal impact of brain regions onto each other might be of use.

In the present study we present a novel approach which consists of the direct estimation of Kuramoto coupling parameters (*K*) from empirical data (section 2.2). Statistical analysis is designed (section 2.4) to assess significance based not on individual coupling parameters, but on whole sets of couplings, which is in line with the underlying assumptions. The focus, therefore, shifts away from spatial localization toward modifications of the dynamics of the brain as a system. We use an exploratory data set to detect dependencies of *K* on clinical severity. These specific hypotheses are then tested in an entirely independent larger test data set, which underwent identical preprocessing. Specifically, within the initial exploratory analysis, we apply this method to intrinsic brain networks (IBNs) in a collective of patients with major depressive disorder (MD) and matched healthy controls, and compare it to three other measures (section 2.3): (i) functional connectivity via partial correlations (*FC*), (ii) phase synchrony (*PS*), and (iii) parameters of a first order auto-regressive model (*AR*). We then test if significant findings on *K* can be recovered in the independent test data set. We hypothesize that *K*s show wide spread alterations with severity of pathology. By a set of simulations (section 4, Appendix A) we test the ability of our method to recover parametric dependencies and delineate scenarios in which parametric dependencies of *K*s on external variables are accessible to the methodology.

## 2. Materials and Methods

### 2.1. Empirical Data of Resting State fMRI in Patients With Major Depressive Disorder and Healthy Controls

#### 2.1.1. Participants

##### 2.1.1.1. Exploratory Data Set

Data was acquired in 25 patients with recurrent MD and 25 age matched control subjects. One patient and one control had to be excluded due to image artifacts, resulting in 24 subjects per group. Mean age in the MD group was 48y [min/max: 23y/79y, 13 female], and 44y [min/max: 26y/68y, 14 female] in the control group. All patients received medication at the time of scanning. Supplementary Table 1 provides details on demographic and clinical characteristics. Participant data have been used in several previous studies (Manoliu et al., 2014; Meng et al., 2014; Ries et al., 2018, 2019). To render results of the IBN determination more robust 25 young healthy control (HC) participants (age 19–32, right handed) were included into the independent component analysis described below. All participants gave informed consent in accordance with the in-house ethics committee of the Klinikum rechts der Isar, TU Munich. Patients were recruited at the psychiatry department of the Klinikum rechts der Isar, TU Munich. Clinical assessment, including DSM IV (American Psychiatric Association, 2000) and Hamilton Rating Scale for Depression (Ham-D, Hamilton, 1960), was performed by two experienced psychiatrists [Structured Clinical Interview for DSM-IV (SCID) inter-rater reliability of 95%]. HCs were recruited by word-of-mouth advertising. MD was the primary diagnosis for all patients, with all of them meeting criteria for a current major depressive episode (MDE) with an average current episode length of 16 weeks (SD = 7), an average Ham-D score of 21.8 (SD = 7.1). The mean duration of MD was 16.72 years (SD = 10.20), the mean number of episodes 6 (SD = 3). Exclusion criteria for patients were psychotic symptoms, schizophrenia, schizo-affective disorder, bipolar disorder, and substance abuse. Exclusion criteria for all participants were pregnancy, neurological or severe internal systemic diseases, and general contraindications for MRI. All patients were medicated (for details see Meng et al., 2014) except for one patient who was free of any psychotropic medication during MRI assessment.

All participants underwent 10 min of rs-fMRI with the instruction to keep their eyes closed and not to fall asleep.

##### 2.1.1.2. Test Data Set

All subjects were included in the study after signing an informed consent form approved by the Investigational Review Board (IRB) at Indiana University School of Medicine and at the Cleveland Clinic Foundation.

One hundred seven medication-free MD subjects and 51 HCs were recruited as part of a study of young adult MD subjects at high and low risk for bipolar disorder. Out of the 107 MD subjects 28 subjects were excluded due to excessive motion, falling asleep during scanning, incomplete or lacking data, and poor data quality. Thirty-five further subjects were excluded from the statistical analysis due to inconsistent imaging parameters, and mild symptoms of mania as assessed by a score of more than 1 on the Young Mania Rating Scale (YMRS). Out of the 51 HCs 10 subjects were excluded due to excessive motion, falling asleep during scanning, incomplete or lacking data, and poor data quality or family history of psychiatric illness. Four further subjects were excluded from statistical analysis due to inconsistent scanning parameters. Data of the extended patient data set without restrictions on YMRS and inconsistent imaging parameters were processed and included into the independent component analysis described below.

Finally, 44 patients (age: mean [min/max] 24y [18y/30y], 33 females) and 37 healthy controls (age: mean [min/max] 24y [18y/30y], 22 females) were included into the statistical analysis. See Supplementary Table 1 for a detailed presentation of demographic and clinical characteristics. A subgroup of the collective was part of a previous investigation (Wohlschläger et al., 2018). Both patients and HCs were paid $25 for screening and $75 for MRI scan. All subjects underwent a detailed structured diagnostic interview—Mini Neuropsychiatric Interview (MINI) that generated a DSM-IV diagnosis (Sheehan et al., 1997). Inclusion criteria for MD were: (1) between 15 and 30 years and able to give voluntary informed consent; (2) satisfy DSM-IV-TR criteria for MD using a structured interview; (3) never met criteria for mania or hypomania; (4) 17-item Ham-D >18 and < 25; (5) Young Mania Rating Scale (YMRS) (Young et al., 1978) score < 10; (6) satisfy safety criteria to undergo an MRI scan; (7) able to be managed as outpatients during the study, ascertained by the following—(i) Clinical Global Severity Scale < 5 i.e., moderately ill, (ii) no significant suicidal or homicidal ideation or grossly disabled.

All participants underwent 6:16 min of rs-fMRI with the instruction to keep their eyes open and to look at a fixation cross.

#### 2.1.2. MRI Data Acquisition

##### 2.1.2.1. Exploratory Data Set

All measurements were performed on a 3T MR scanner (Achieva, Philips, Netherland) using an 8-channel phased-array head coil. T1-weighted anatomical images were obtained from a magnetization-prepared rapid acquisition gradient echo (MPRAGE) sequence (FoV = 240 × 240 mm^2^, 170 slices). FMRI data were obtained from a gradient echo planar imaging (EPI) sequence (TR/TE = 2, 000/35 ms, 32 slices, slice thickness = 4 mm, 300 volumes). For the additional data set of young subjects respective parameters were: T1 (FoV = 480 × 480 mm^2^, 340 slices), fMRI (TR/TE = 2, 007/30 ms, 36 slices, slice thickness = 3 mm; 300 volumes).

##### 2.1.2.2. Test Data Set

Measurements were acquired at Cleveland Clinic Main Campus using 3T Siemens Prisma MR Scanner. T1-weighted anatomical images were obtained from a MPRAGE sequence (FoV = 240 × 256 mm^2^, 160 slices). FMRI data were obtained from an EPI sequence (TR/TE =2, 800/29 ms, 39 slices, slice thickness = 3.5 mm, 132 volumes). To limit the head motion scans at Cleveland Clinic were acquired with subjects fitted with a bite bar.

#### 2.1.3. Preprocessing

During preprocessing particular care was taken to address physiological as well as movement artifacts. Effects of heart beat and breathing were quantified from the data using Physiologic Estimation by Temporal ICA (PESTICA) (Beall and Lowe, 2007), and a physiologic noise removal tool, RETROICOR (Glover et al., 2000). During this step instantaneous effects of heart beat and respiration are corrected for. Estimates of the cardiac and respiratory rates can be retrieved. There were no significant group differences in both measures. Because it has been shown that magnitude of cardiac and respiratory rate can have delayed effects within the BOLD signal (Birn et al., 2008; Chang et al., 2009) respective regressors were calculated and accounted for within the subsequent procedures (see below). Movement correction was optimized with slice-based realignment using slice-oriented motion correction (SLOMOCO) (Beall and Lowe, 2014). No significant group differences in volume-wise or slice-wise mean motion were detected (see Supplementary Table 1). Further preprocessing steps included coregistration to the T1 image, slice time correction, spatial normalization, and spatial smoothing with the full width at half maximum (FWHM) of the Gaussian filter 8 × 8 × 8 mm^3^ (SPM12, https://www.fil.ion.ucl.ac.uk/spm/).

#### 2.1.4. IBN Time Course Preparation

Exploratory and test data sets of the preprocessed data were each entered into independent component analyses (ICA) and each separated into 75 spatially independent components (Calhoun et al., 2001) based on the Infomax-algorithm and implemented in the fMRI Toolbox (GIFT, http://www.icat.sourceforge.net) which was run 20 times through ICASSO to ensure stability of the estimated components. For both data sets group average components were back-projected on the single-subject data. Via multiple spatial regression 20 of the 75 independent components in the exploratory data set and 28 of the 75 independent components in the test data set were identified as neuronally meaningful IBNs with correlation coefficients above 0.15 to the spatial templates of the IBNs as described in Allen et al. (2011). The IBNs are presented in the Supplementary Figures 1, 2. Time courses from these IBNs for each subject were corrected for effects of white matter signal, and signal from the cerebrospinal fluid, and additionally for effects of the respiratory (Birn et al., 2008), and the cardiac (Chang et al., 2009) response functions, by regression. The latter two functions compensate for delayed effects of variations in respiratory and breathing rates on the BOLD signal. In order to select a frequency band affected by dynamical changes in the patient group, metastability was calculated for a range of frequency bins (Ries et al., 2019) as described in the Supplementary Material (section 1.3) (Figure 3). Frequency bin 3 displayed significant reduction in metastability in the patients. Based on this finding, time courses were bandpass filtered to a narrow frequency band of 0.05–0.075 Hz in preparation for a subsequent Hilbert transformation (Córdova-Palomera et al., 2017) using a Butterworth filter of order 7. The *FC* and *AR* measures are not based on phase analysis, therefore, the frequency range does not have to be that strongly reduced. Here the less stringent, commonly used frequency band of 0.01–0.1 Hz (Zang et al., 2007) was chosen as band pass filter.

### 2.2. Calculation of Kuramoto Coupling Coefficients

This section describes the methodological concepts which we will use to analyze the coupling behavior in our data sets. Table 2 gives an overview of the most important notation used in the following sections.

**Table 2:** Notation.

**Notation**	**Meaning**
**X**	Bold capital letters indicate matrices
**x**	bold small letters indicate vectors
*X*,*x*	Non bold capital or small letters indicate real numbers
𝕏	Three dimensional matrix
**x**_*i*_	*i*−th row of a matrix **X**
*X*_*i,j*_	(*i,j*)−th entry of a matrix **X**
*x*_*i*_	*i*−th entry of a vector **x**
*s*	Number of subjects
*r*	Number of IBNs per subject
*T*	Number of measure points in the recording
*K*	Notation for the measure “Kuramoto coupling parameters” for empirical data analysis
𝕂^o^, **K**^o^, $K_{i,j}^{\text{o}}$	Original random coefficient matrices/matrix/matrix entry for the simulations
𝕂^c^, **K**^c^, $K_{i,j}^{\text{c}}$	Manipulated coefficient matrices/matrix/matrix entry with induced correlations on *score*
𝕂^res^, **K**^res^, $K_{i,j}^{\text{res}}$	Resulting Kuramoto coefficient matrices/matrix/matrix entry calculated with our model
ω_*i*_	Eigenfrequency of the *i*−th oscillator/IBN
*d*	Overall coupling parameter
*n*	Noise level weight
**N**, **N**_s_, **N**_as_	Correlation patterns
**M**, **M**_in/out_	Individual coefficient weight matrix (“mask matrix”)

#### 2.2.1. Kuramoto Model

First, we consider the Kuramoto model (KM) (Kuramoto, 1975; Acebrón et al., 2005). This is a system of ordinary differential equations (ODEs) describing the temporal change of the phases φ_1_, …, φ_*r*_ of *r* oscillators, which are coupled by the sine of their phase differences:


(1) $$\begin{array}{l} {\dot{\varphi }}_{i}\left(t\right)=\frac{\partial \varphi_{i}\left(t\right)}{\partial t}=\omega_{i}+\frac{C}{r}\overset{r}{\underset{j=1}{\sum }}K_{i,j}\sin\left(\varphi_{j}\left(t\right)-\varphi_{i}\left(t\right)\right)=f\left(t,\varphi_{i}\left(t\right)\right)\\ \;i=1,\ldots ,r. \end{array}$$

Here, φ_*i*_(*t*) is the phase angle of the *i*–th oscillator at time point *t* and ω_*i*_ is the eigenfrequency of the i-th oscillator. The only parameters in this model are the coupling coefficients *K*_*i,j*_ describing the connection between oscillator *i* and *j*. In this model couplings between each and every oscillator are considered, which matches our assumption of wide-spread effects and simultaneous involvement of all IBNs. The coupling strength *C* can be set to 1 since it is an equal scaling for the *K*_*i,j*_ parameters. Note, that the model can attain different forms. The choice of this form is discussed in the Supplementary Material (section 2.1).

Each subject in our data set has *r* time courses *x*(*t*) representing the activity in *r* IBNs. The use of this model for our time series data *x*(*t*) first requires the extraction of the instantaneous phases φ(*t*) for each time course.

#### 2.2.2. Hilbert Transform

The Hilbert transform (Hahn, 1996) denoted by *H*{*x*(*t*)} returns a version of the original time series shifted by $\frac{\pi }{2}$. Considering the analytical signal *x*_*a*_(*t*)=*x*(*t*)+*i*·*H*{*x*(*t*)}, we can then represent the time series in an amplitude-phase representation.


(2) $$\begin{array}{l} x\left(t\right)=a\left(t\right)\cdot \cos\left(\varphi \left(t\right)\right) \end{array}$$

with the instantaneous amplitude *a*(*t*)=|*x*_*a*_(*t*)| and the instantaneous phase φ(*t*)=∠*x*_*a*_(*t*).

#### 2.2.3. Numerical Solution Method

The usual way of utilizing an ODE model such as the KM, is to set the model parameters (in this case the *K*_*i,j*_) suitable for the domain application and subsequently calculate a solution that fulfills the model equations (i.e., the functions φ_1_, …, φ_*r*_) with a numerical solver. This has been done previously in Neuroscience (Stramaglia et al., 2017)—also for the Kuramoto model (Schmidt et al., 2015). There exist many numerical approximation methods. One of the most basic approaches is Euler's method (Epperson, 2013). The approximation of the solution is calculated step-wise according to the following rule


(3) $$\begin{array}{l} \varphi_{i}\left(s+1\right)=\varphi_{i}\left(s\right)+f\left(s,\varphi_{i}\left(s\right)\right). \end{array}$$

with the iteration steps *s* and *s* + 1 and a step size *h* chosen as 1. Discussion about this choice and also the choice of the Euler method as a numerical solution method can be found in the Supplementary Material (section 2.2).

In this work, we are already given phase courses from the recordings (i.e., the functions φ_1_, …, φ_*r*_). The time courses in our experiments are filtered to a very narrow frequency band. Therefore, we can model the eigen-frequencies ω_*i*_ as the mean frequency $\overset{-}{\omega }$ of the respective frequency band. Another option would be to estimate each eigen-frequency from the largest peak in the frequency profile of each time course via fast Fourier transform (FFT). Our codes provide both options. Unless mentioned otherwise, all our experiments were conducted using a fixed mean eigen-frequency. Given all phase and eigen-frequency values, we are able to choose a reverse engineering approach. We assume that the KM can describe synchronization or coupling respectively of our given data and utilize formula (3) to calculate the KM parameters *K*_*i,j*_ which optimally fit our data.

#### 2.2.4. Goal

Our goal is the estimation of the coupling coefficients *K*_*i,j*_ of model (1) considering the given phase courses (φ_1_(*t*), …, φ_*r*_(*t*), *t*=1, …*T*) and eigen-frequencies (ω_*i*_) of the IBN time series. The coefficients may be interpreted as the coupling strengths between the time series and, therefore, between the IBNs of a subject. We proceed as follows.

#### 2.2.5. Linear Equation System

By transforming the time series *x*_*i*_(*t*) of each IBN of a participant using the Hilbert transformation, we get an amplitude-phase representation of each time series


(4) $$\begin{array}{l} x_{i}\left(t\right)=a_{i}\left(t\right)\cdot \cos\left(\varphi_{i}\left(t\right)\right)\;t=0,\ldots ,T-1. \end{array}$$

This way we obtain the actual time course of the phases φ_*i*_(*t*) of the time series *x*_*i*_(*t*), *i*=1, …, *r*. So instead of simulating the synchronization of initial phase values of a subject's time series, we assume that synchronization of the phases is explainable by the above Kuramoto model (1). Considering the phase values φ_*i*_(*t*), *t*=0, …, *T* − 1 of a single time course of a single subject and plugging in those phase values into Equation (3) leads to linear equations of the form


(5) $$\begin{array}{l} \varphi_{i}\left(s+1\right)=\varphi_{i}\left(s\right)+\omega_{i}+\frac{1}{r}\overset{r}{\underset{j=1}{\sum }}K_{i,j}\sin\left(\varphi_{j}\left(s\right)-\varphi_{i}\left(s\right)\right),\\ \text{ \#x000A0; }s=0,\ldots ,T-2. \end{array}$$

In our exploratory data set, we have *T* = 300 measure points in the recording and each of the *s* = 24 subjects per group has recordings for *r* = 20 IBNs. Thus, we obtain 299 (# time steps) equations for 20 (# IBNs) unknown variables *K*_*i,j*_, *j*=1, …, 20 for each IBN of each subject. This results in total in 400 coefficients *K*_*i,j*_, *i, j*=1, …, 20 for one subject. Note again, that time steps are labeled as *s*=0, 1, 2, …, 299 corresponding to iterations while the values correspond to time points *t*=0, 2, 4, …, 598 in seconds. The equations can be rearranged to the form of a linear equation system (LES)


(6) $$\begin{array}{l} {\text{S}}_{i}\cdot {\text{k}}_{i}={\text{b}}_{i}, \end{array}$$

with ${\text{S}}_{i}\in {\mathbb{R}}^{299\times 20}$, ${\text{k}}_{i}\in {\mathbb{R}}^{20}$, and ${\text{b}}_{i}\in {\mathbb{R}}^{299}$. The system matrix **S**_*i*_ will, however, have one zero column, which corresponds to the coefficient *K*_*i,i*_. We have to eliminate this column in order to obtain a system matrix with full rank. Accordingly, we reduce the number of unknowns by not solving for *K*_*i,i*_, but determining it instead. As it represents the coupling between an IBN to itself, we simple set the value to 1 (any constant would suffice). Thus, we will determine 380 coefficients per subject. Details about the entries of **S**_*i*_ and **b**_*i*_ as well as the derivation of the entries can be found in the Supplementary Material (section 2.3).

#### 2.2.6. Solving the LES

Since the LES (6) is over-determined, i.e., the number of equations is larger than the number of variables, we can not simply invert the non-squared system matrix **S**_*i*_. We solve the over-determined LES optimal with respect to the ℓ_2_ norm by building the *normal equations* (Gauß, 1809; Abdulle and Wanner, 2002).


(7) $$\begin{array}{l} \underset{{Ŝ}_{i}}{\underset{︸}{{\text{S}}_{i}^{\text{T}}{\text{S}}_{i}}}\cdot {\hat{\text{k}}}_{i}=\underset{{\text{b}}_{i}}{\underset{︸}{{\text{S}}_{i}^{\text{T}}\cdot {\text{b}}_{i}}}. \end{array}$$

The symmetric matrix ${\hat{\text{S}}}_{i}\in {\mathbb{R}}^{19\times 19}$ is regular and can, therefore, be inverted to obtain a unique solution ${\hat{\text{k}}}_{i}\in {\mathbb{R}}^{19}$, where it holds


(8) $$\begin{array}{l} {\left\|{\text{S}}_{i}\cdot {\hat{\text{k}}}_{i}-{\text{b}}_{i}\right\|}_{\ell_{2}}^{2}=\underset{\text{x}\in {\mathbb{R}}^{19}}{\min}{\left\|{\text{S}}_{i}\cdot \text{x}-{\text{b}}_{i}\right\|}_{\ell_{2}}^{2} \end{array}$$

Derivations of the normal equations to yield the optimal solution in the sense of Equation (8) are given in the Supplementary Material (section 2.4). In order to solve the equations simultaneously for all IBNs of one subject, we build a block diagonal equation matrix, and append the inhomogeneity terms resulting in


(9) $$\begin{array}{l} \text{S}=\left(\begin{array}{cccc} {\text{S}}_{1} & 0 & \cdots & 0\\ 0 & {\text{S}}_{2} & \cdots & 0\\ \vdots & \vdots & \ddots & \vdots \\ 0 & 0 & \cdots & {\text{S}}_{20} \end{array}\right) \end{array}$$

(10) $$\begin{array}{l} \text{b}=\left(\begin{array}{c} {\text{b}}_{1}\\ {\text{b}}_{2}\\ \vdots \\ {\text{b}}_{20} \end{array}\right) \end{array}$$

We calculate the solution $\hat{\text{k}}\in {\mathbb{R}}^{380}$ of the LES


(11) $$\begin{array}{l} {\text{S}}^{\text{T}}\cdot \text{S}\cdot \hat{\text{k}}={\text{S}}^{\text{T}}\cdot \text{b} \end{array}$$

by a simple matrix inversion


(12) $$\begin{array}{l} \hat{\text{k}}={\left({\text{S}}^{\text{T}}\cdot \text{S}\right)}^{-1}\cdot {\text{S}}^{\text{T}}\cdot \text{b} \end{array}$$

Finally, we set the resulting Kuramoto coupling coefficients for one subject ${\text{k}}^{\text{res}}:=\hat{\text{k}}$. We perform these steps for each subject which provides us with 380 coefficients $K_{i,j}^{\text{res}}$, *i, j*=1, …, 20, *i* ≠ *j* per individual. The vector **k**^res^ can be reshaped to a Kuramoto coupling matrix **K**^res^ of size 20 × 20 by putting the coefficient estimating the coupling between IBN *i* to IBN *j* in the (*i, j*)−th entry and filling up the diagonal with ones. This can nicely be visualized by a heat map (see Supplementary Figure 4 for an example visualization). Gathering all Kuramoto coupling coefficient for all subjects in a group results in a three dimensional matrix object of size *s* × *r* × *r*, which we term *K*^res^. This object contains the calculated Kuramoto coupling coefficient, which are given the marker name *K* in Introduction (section 1), Results (section 3.1) and Discussion (section 5) in the context of the empirical data sets.

All calculations were implemented in Matlab R2018a^1^. Scripts are available via the following link: https://doi.org/10.6084/m9.figshare.13352399.

### 2.3. Calculation of Reference Coupling Measures

#### 2.3.1. Partial Correlations (*FC*)

Time course data were filtered to a frequency band of 0.01–0.1 Hz (Zang et al., 2007). Partial correlations between each and any of the IBNs were calculated from the time courses of all IBNs using Matlab.

#### 2.3.2. Phase Synchrony (*PS*)

Time course data were filtered to a frequency band of 0.05–0.075 Hz (as in the calculation of *K*) in preparation for the subsequently conducted Hilbert transformation (Córdova-Palomera et al., 2017). *PS* were calculated pairwise from the phase time courses between each and any of the IBNs according to


(13) $$\begin{array}{l} PS_{i,j}=\text{med}\left(IPS_{i,j}\left(t\right)\right)=\text{med}\left(\cos\left(\varphi_{j}\left(t\right)-\varphi_{i}\left(t\right)\right)\right) \end{array}$$

Here, med(·) is the median function across time applied to the instantaneous phase synchrony *IPS*(*t*) (Zarghami et al., 2020) and φ_*i*_(*t*) is the phase angle of the *i*–th oscillator at time point *t* given in rad.

#### 2.3.3. Coefficients of a First Order Auto-Regressive Model (*AR*)

Time course data were filtered to a frequency band of 0.01–0.1 Hz (Zang et al., 2007). Calculation of the auto-regressive coefficients, including auto-regression within one and the same time course, were performed using the scripts available from Liégeois et al. (2019) according to their Equation (1):


(14) $$\begin{array}{l} x\left(t\right)=AR\times x\left(t-1\right)+\epsilon \left(t\right) \end{array}$$

### 2.4. Statistical Analysis

#### 2.4.1. Set-Level Statistics

We were particularly interested in wide spread changes of *K*s and the control measures across the whole brain. We therefore tested whether the number of individual correlations depending on a regressor (Ham-D) were likely to occur by chance. To this, we compared the sizes of sets containing couplings with statistically significant dependencies on the regressor against the distribution of set sizes derived from random permutations of *K*-values.

**Figure 2:**
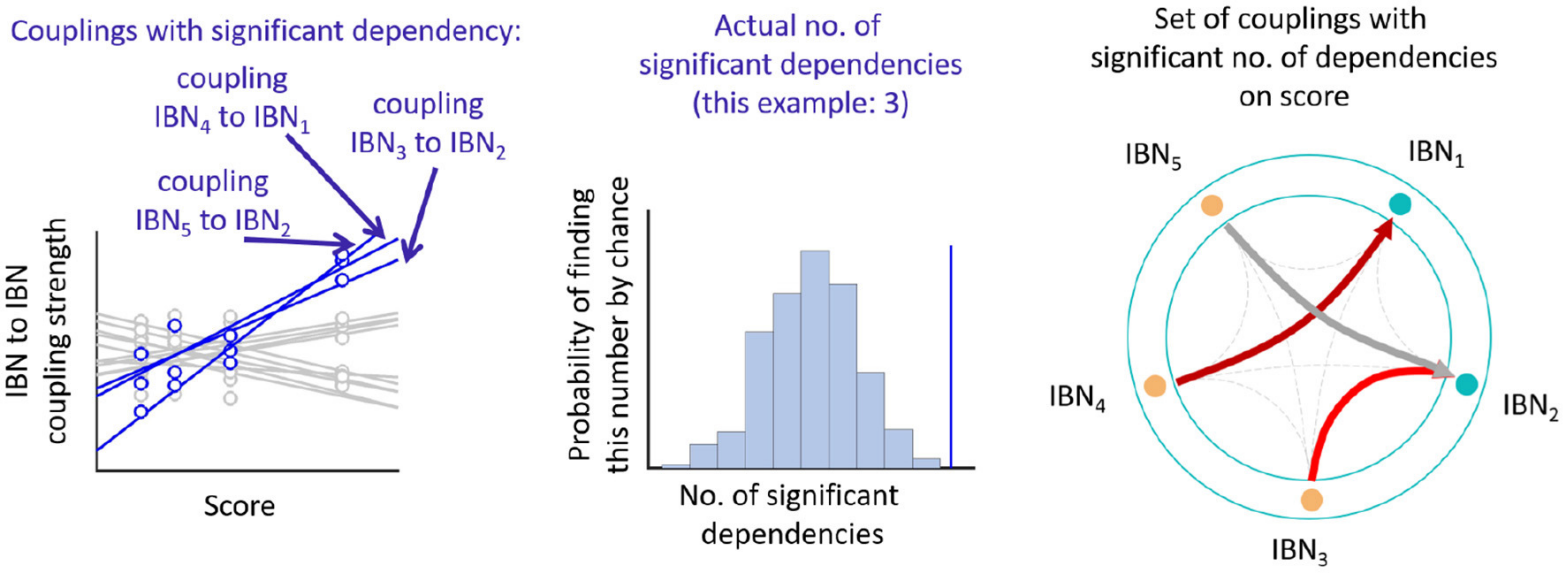
Analysis pipeline on empirical data. **(Left)** From correlations of all individual coupling parameters to a parametric score across subjects, moderately significant correlations (blue) vs. non-significant correlations (gray) are detected. **(Middle)** The number of these correlating couplings (“set size”) is compared to the distribution of the set sizes derived from permutations of the couplings. **(Right)** Sets of couplings reaching significance are displayed.

In detail, we assessed the significance of sets of *K*_*i,j*_*s* showing moderate associations to a parametric regressor per group via the number of these association. We calculated Spearman correlations per individual coupling to a given regressor, and counted the number of significant correlation at a threshold of *P*_*u*_ < 0.05, uncorrected for multiple comparisons. Subsequently, all couplings were permuted within each subject of the group and we repeated the correlation procedure yielding a number of chance correlations and, therefore, chance set sizes at the threshold of *P*_*u*_ < 0.05. By repeating this procedure we produced a distribution of the number of chance set sizes, to which we compared the actual set size (see Figure 2). We generated a *P*-value from the percentage of chance set sizes larger than the actual set size. The script accounts for the fact, that matrices can be symmetric (*FC*, *PS*) or non-symmetric (*K*, *AR*). A number of 500 permutations yielded stable results for the *P*-values.

We performed similar tests on the set-level for finding sets of significant couplings versus zero and sets of significant couplings showing group differences, by replacing the correlation procedure with Wilcoxon signed rank and Wilcoxon rank sum test, respectively.

Data deviating more than two standard deviations from the mean were regarded as outliers. All procedures were implemented in Matlab including parts of the script *sig_permtest.m* (http://commdetect.weebly.com/).

Corrections for multiple comparisons was necessary, because we performed correlations on two regressors (Ham-D, Age) in three versions (bi-directional, positive, negative) in the patient group, amounting to a Bonferroni factor of 6. In the control group we only analyzed the age regressor in the three versions, amounting to a Bonferroni factor of 3. In all other tests of couplings versus zero or between groups we corrected the threshold for significance by a Bonferroni factor of 3 for the three directions of the test always performed. *P*-values reported for the test data set are not corrected for multiple comparisons.

In the exploratory data set, dependencies of coupling sets with age were found for *PS* in patients as well as healthy controls [MD: bi-directional (*i*): *P*_*u*_=0.004, positive (*ii*): *P*_*u*_=0.022, negative (*iii*): *P*_*u*_=0.008; HC: bi-directional (*i*): *P*_*u*_=0.036, positive (*ii*): *P*_*u*_=0.046, negative (*iii*): *P*_*u*_=0.092, uncorrected for multiple comparisons]. No such sets were found for *K* and *AR*. Therefore, all set-level statistics for *FC* and *PS* were corrected for age by regressing out age from each individual coupling. There was no significant dependence on sex for any coupling measure.

We investigated correlations of *K* with mean head motion and cardiac rate. In the patient group of the exploratory data set we detected a correlation of head motion with sets of *K*s. This is discussed detail in Supplementary Material (section 4.1). There were no other significant correlations of *K* with movement or cardiac rate.

#### 2.4.2. Phase Randomized Surrogates

Significant dependence of the coupling measures *K* and *PS* on regressors was additionally assessed by using phase randomized surrogates to eliminate any estimation bias. Time course phases were randomized while preserving their power spectra by the following steps (Ponce-Alvarez et al., 2015): (i) each time course underwent Fourier transformation, (ii) the phase values were replaced by values from a random uniform distribution between −π and π, and (iii) in order to return to the time domain an inverse Fourier transform was applied. *K* and *PS* calculation was applied to the phase randomized data sets. Significance of set-level correlation was tested against 500 phase randomizations.

## 3. Results

### 3.1. Results on Empirical fMRI Data in Patients With Major Depression and Healthy Controls

#### 3.1.1. Sets of *K*s Show Particular Dependence on Disease Severity

We wanted to see if there were sets of specific *K*s exhibiting group differences or parametric dependencies in the exploratory data set. We used a permutation approach to estimate the probability of the number of couplings showing moderate dependence on either group or a parametric regressor occurring by chance (see Figure 2). This approach allows for detecting parametric dependencies with opposite signs in different couplings. The tests were, therefore, performed separately (i) irrespective of the direction of associations between couplings and parametric regressor or group, (ii) for positive associations, or (iii) negative associations.

With regards to group differences, none could be detected from the *K*s. *FC* provided a set of couplings being at trend (*P*_*c*_=0.063) in the contrast of type (*ii*), i.e., they displayed higher values in the patient group than in controls (see Supplementary Figure 8, section 4.2). No other significant or trending sets were found.

Contrasting to the lack of overall group difference, *K*s yielded sets of couplings displaying significant parametric dependence on the regressor of interest in the patient group.

**Figure 3:**
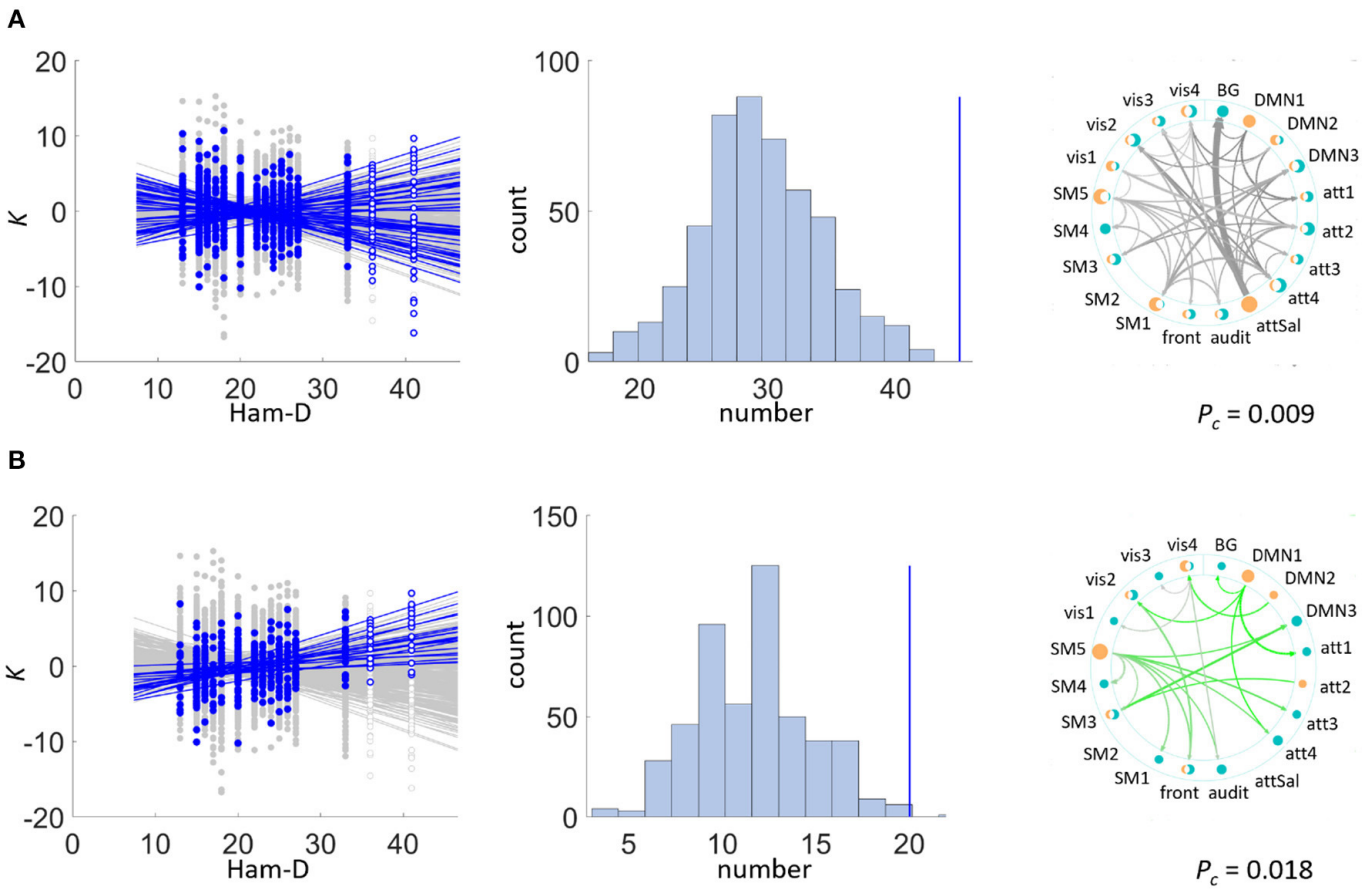
Set-level results. Sets of couplings of statistically significant size were detected in the exploratory data set within the patient group: *K* significantly depended on Ham-D in **(A)** the bi-directional test, mainly driven by **(B)** positive correlations. Left side plots depict the individual significant correlations of couplings on the regressor in blue and in gray otherwise. Note that indicated regressions in this figure were fitted to data excluding outliers (non filled markers). Middle plots depict the chance distribution on set sizes, with the actual data indicated by the blue vertical line. The right plot displays those connections constituting the significant set. Color varies with direction of correlation: bi-directional (grey), positive (green), line width scales with correlation coefficient ρ. Dots marking the IBNs are two-fold for outbound (orange) and inbound (teal) couplings, scaling in size with overall coupling strength toward all other IBNs. **(A)** Patient group: *K* vs. Ham-D. **(B)** Patient group: *K* vs. Ham-D (positive dependence).

Sets of *K*s were significantly depending on the Ham-D score in the patient group (see Figures 3A,B and Table 3, showing the uncorrected *P*-values). The dependence was mainly driven by a positive correlation of type (*ii*) (*P*_*c*_=0.012), meaning that *K*s increased with higher disease severity, but a set including both ways of dependence, i.e., of type (*i*) was also significant (*P*_*c*_=0.006). The set with negative dependence on Ham-D of type (*iii*) was not significant (*P*_*c*_=0.096). We checked for set-level significance of the dependence on the Ham-D score when the eigenfrequencies in equation (1) are calculated from the data. This method yielded very similar results (*i*) *P*_*c*_=0.012, (*ii*) *P*_*c*_=0.036, (*iii*) *P*_*c*_=0.11.

**Table 3:** Set level statistics.

	**MD**		**HC**	
**EXPL**.	**Ham-D**	**Age**	**Age**	
	**All (pos.neg.)**	**All (pos.neg.)**	**All (pos.neg.)**	
*K*	**0.001** (**0.002**/0.016)	n.s. (n.s./n.s.)	n.s. (n.s./n.s.)	
	**<0.001** (**0.004**/** <0.001**)	n.s. (n.s./n.s.)	n.s. (n.s./n.s.)	†
*FC*	n.s. (n.s./n.s.)	n.s. (n.s./0.080)	**<0.001** (**0.003**/**0.005**)	
*PS*	n.s. (n.s./n.s.)	**0.004** (0.022/0.008)	0.036 (0.046/0.092)	
	n.s. (n.s./n.s.)	**<0.001** (0.018/** <0.001**)	0.046 (0.022/n.s.)	†
*AR*	n.s. (n.s./n.s.)	n.s. (n.s./n.s.)	n.s. (n.s./n.s.)	
**TEST**				
*K*	0.006 (0.002/0.12)			
	0.022 (0.032/0.19)			†

*P-values, not corrected for multiple comparisons, from permutation testing indicating the significance of the size of a set appearing by chance, for bi-directional test (“all”), or including only positive (“pos.”) or only negative (“neg.”) correlations. For the exploratory data sets P-values surviving multiple comparisons correction, with Bonferroni factors 3 and 6 for HC and MD, respectively, are indicated in bold print. FC and PS were corrected for age in all tests, except for vs. age itself. For the test data set the table provides the P-values derived from re-assessing the significant or close to significant dependencies from the exploratory data set in the test data set. Statistical testing was performed against measures calculated from phase randomized surrogates. n.s., not significant*.

All other analyses of *FC*, *PS*, and *AR* in patients and controls yielded no significant results in the dependence on Ham-D.

A significant negative correlation was found between the coupling *K* from the salience network onto the basal ganglia network (*P*_*c*_=0.002) to Ham-D after rigorous Bonferroni correction for 380 multiple comparisons. This coupling was the only one showing a significant dependence on a score under investigation.

The re-assessment of set dependence on Ham-D in the test data set shows that the association of *K* to this external regressor can be recovered in the test data set.

## 4. Validation

The *K* couplings did not significantly differ from zero. Nevertheless, on a set level, dependencies to clinical severity scores could be detected in two independent empirical data sets. As an additional validation of the capabilities of our novel method, we conducted experiments with synthetically generated data. More specifically, we simulated phase courses, where we induce a dependence in the data generating coupling parameters on an independent score. Our hypothesis is, that our model should be able to detect these dependencies in the generated data. The purpose of these experiments is not to prove our method superior in comparison to other methods, but they should serve as a proof of concept, that induced dependencies can be recovered by our method.

### 4.1. The Simulation Model

We generate a synthetic data set for *s* subjects with *r* IBNs and *T* measure points each. We utilize the Kuramoto model to simulate the data, but we alter model (1) in various aspects to fit our purpose of generating phase courses. The simulated phase courses for one subject are the solution functions of the following Kuramoto model:


(15) $$\begin{array}{l} {\dot{\varphi }}_{i}\left(t\right)=\frac{\partial \varphi_{i}\left(t\right)}{\partial t}\\ \;=\omega_{i}+\frac{d}{r}\overset{r}{\underset{j=1}{\sum }}K_{i,j}\cdot M_{i,j}\cdot \sin\left(\varphi_{j}\left(t\right)-\varphi_{i}\left(t\right)\right)+n\cdot \varepsilon_{i}\left(t\right)\\ \;i=1,\ldots ,r. \end{array}$$

As shown several parameters are included in the model now. First, we include eigen-frequencies ω_*i*_ for the oscillators. These are the driving forces hindering synchronization. The coupling coefficients *K*_*i,j*_ are the second forces determining synchronization behavior between each two oscillators *i* and *j*, i.e., **K** ∈ ℝ^*r*×*r*^. The parameter *d* is a positive weight for the coupling coefficients, which acts equally on all coefficients and can be seen as an overall coupling strength. The additional weight *M*_*i,j*_ acts individually on each single coefficient, i.e., **M** ∈ ℝ^*r*×*r*^. Furthermore, we include noise ε_*i*_(*t*) in our model which is also weighted with an intensity level *n*.

To obtain simulated phase courses, we have to solve the system of ODEs. As the numerical solver of the ODE system, we choose not to work with the same as when calculating the coefficients (i.e., Euler's method). This prevents to simply get out what we put in. The solver used for the simulations is the classical Runge-Kutta algorithm (Schwarz and Köckler, 2011) also called RK-4. Providing initial values φ_*i*_(0), *i*=1, …, *r*, the Runge Kutta method iteratively yields the phase courses. A detailed formulation of the RK-4 method is given in the Supplementary Material (section 3.1).

### 4.2. Simulation Procedure

**Figure 4:**
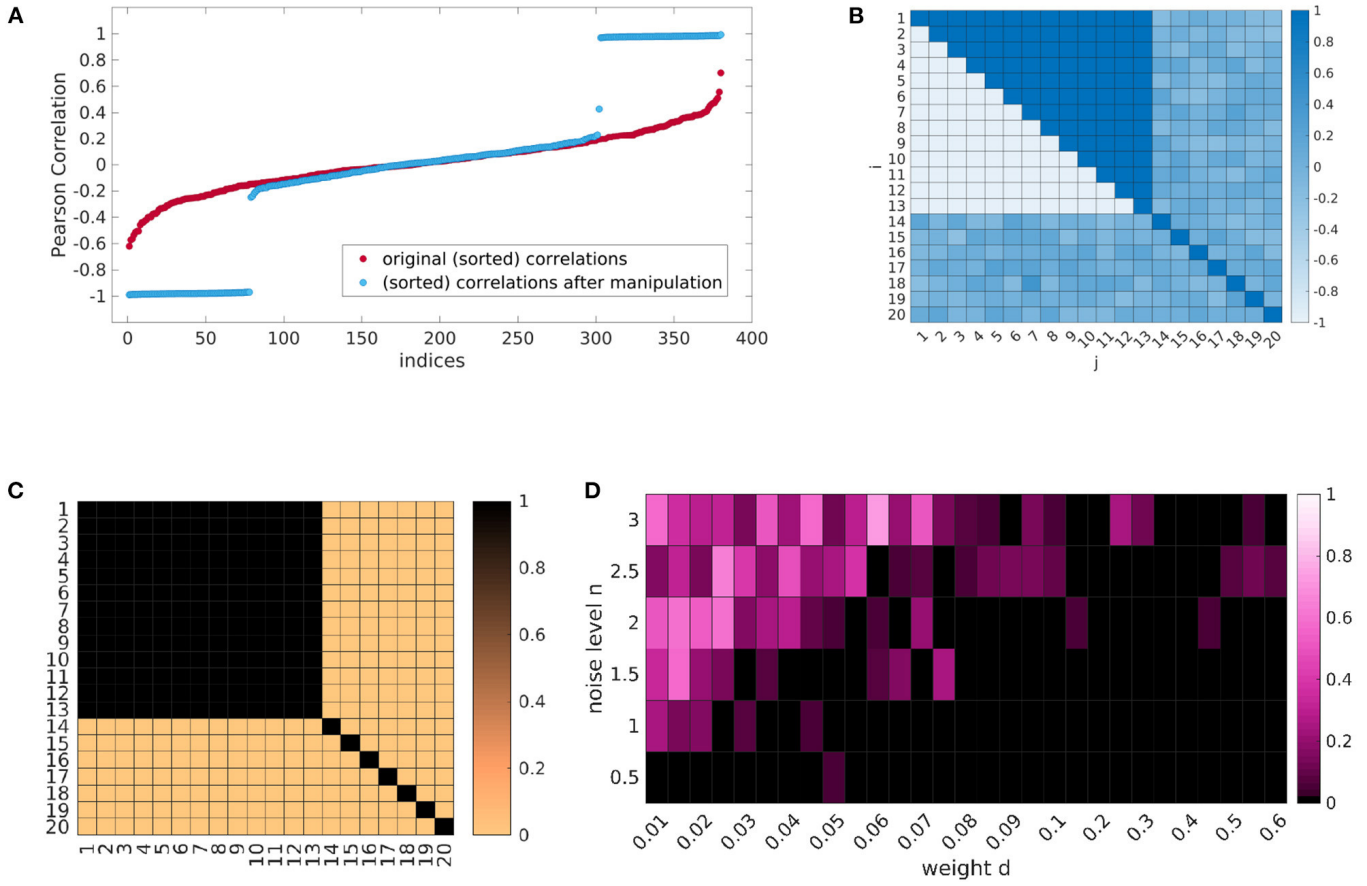
One exemplary result of the simulation procedure. By inserting dependencies on the data generating coupling coefficients **(A)** we can generate a certain correlation pattern **(B)**. Emphasizing the influence of the significant couplings in the data generating process **(C)** allows to retrieve the dependencies with our method **(D)**, when the noise to data ratio is in a certain range. **(A)** Correlations in ascending order for the random initial coefficients and after explicit insertion of parametric dependence. **(B)** Correlation pattern. **(C)** Weight matrix M. **(D)** Median *P*-values over the six runs for all (*n, d*) combinations. (P < 0.05 black).

For best possible comparability, we generate 20 time courses with 300 time points for 24 subjects. Therefore, we have to provide our data generating pipeline with the parameters as explained above. This includes matrices **Ω** ∈ ℝ^20×24^ and $\Phi_{0}\in {\mathbb{R}}^{20\times 24}$ containing the 20 eigenfrequencies and initial phase values for each subject—which are both randomly initialized—, the weight matrix **M** ∈ ℝ^20×20^, and the weights *d* ∈ ℝ^+^ and *n* ∈ ℝ^+^ for the coefficients and noise. We randomly initialize coupling coefficient matrices **K** ∈ ℝ^20×20^ and an independent score value *s* ∈ ℝ (representing the Ham-D) for each subject. Across subjects, the coefficients will not be significantly correlated with this independent score, but we can manipulate each subjects coefficient matrix, such that a certain portion of the coefficients shows very high positive or negative correlation (see Figure 4A). By inserting dependencies on **s** for specific coefficients, this results in a correlation pattern, as can be seen in Figure 4B, where coefficients between regions from 1 to 13 show distinct correlations compared to others. The resulting manipulated coefficient matrices for all subjects *K*^c^ ∈ ℝ^24×20×20^ are then also provided for the data generating procedure. An example of a generated phase course can be seen in Supplementary Figure 5.

For the 24 × 20 × 300 generated phase course data a Kuramoto coupling coefficient matrix is calculated for each subject with our Kuramoto coupling estimation model, which results in 24 matrices of size 20 × 20 (*K*^res^). To account for the randomness in the initialization of **Ω** and **Φ**_**0**_, we repeat the simulation pipeline for six different random phase and eigenvalue initializations and perform evaluations in account of these six runs, i.e., medians of *P*-values. We equidistantly set *d* and *n* within a limited value ranges and make calculations with all combinations of theses two parameters while keeping the other parameters fixed. The simulation process is quite run time intensive, therefore, we choose the number of runs to stay within a moderate run time but at the same time account to a certain degree for the randomness of the initialization. For the cluster permutation test on the resulting *K*^res^, bi-directional correlations were considered with a number of 100 permutations.

### 4.3. Exemplary Result on Simulated Data

One result can be seen in Figure 4D. Each cell of the heatmap shows the median *P*-value of the cluster permutation test over the six runs for one combination of (*n, d*). The correlation pattern of the data generating coupling coefficients corresponds to Figures 4A,B, respectively. The weights in **M** are chosen as shown in Figure 4C, i.e., couplings are only considered in the data generation, if they show a significant correlation. As we can observe, the induced dependence on the independent score **s** can indeed be recovered, when the relation of noise to coupling is in a certain range.

In Appendix A, the full simulation design, parameter choices, pipeline, and results are explained in detail. Additional results are also given in the Supplementary Material.

## 5. Discussion

In the present study we showed that Kuramoto coupling parameters estimated from empirical data relate to a clinical scores indicating disease severity in patients with major depressive disorder. The findings acquired from the *K*-values differ qualitatively from findings with other methods for quantification of functional connectivity and, therefore, rather provide complementary information. While the variability within single *K*-values is minor, significantly larger sets of *K*s relate to external scores. The findings are in line with the initial hypothesis which puts wide-spread coupling changes into context with chemical connectivity alterations at the synapses. The data provide an initial indication that parametric changes of the *K*-values can be discussed in context of pathological alterations of brain function in major depressive disorder (MD). By analyzing artificial data with a ground truth of parametric dependence in the couplings, we prove the ability of our analysis pipeline to recover this dependence within a reasonable parameter space of the model.

A number of recent studies highlight the explanatory power of following the trajectories of fMRI co-activation patterns from time point to time point via analysis of inter-regional connectivity measures (Gu et al., 2015; Braun et al., 2018; Liégeois et al., 2019). Gu et al. (2015) analyze how activity spread along a known structural connectome in a step-wise re-iterated way favors easy to reach states of co-activation patterns over hard to reach states. While this analysis excludes variability due to the impact of chemical transmission in activity flow, it demonstrates that co-activation states observed from fMRI can, in principle, be tracked back to the magnitude of activity flow among the whole set of brain areas from one iteration step to the next. Relating to the latter concept, Liégeois et al. (2019) show in a recent study that parameters estimated for the first order auto-regressive model from fMRI data possess a much higher capacity of explaining variance in behavioral data than a static model. For this, they used a large data set of resting state fMRI data and behavioral scores from the Human Connectome Project. Similarly in dementia, analysis of dynamic fluctuations yields more specific results than analysis of static functional connectivity (Moguilner et al., 2021). Following an alternative approach, parameters of the Ising model can be estimated from empirical data (Nguyen et al., 2017) in an application which is particularly apt for processes on the neuronal level based on binary processes of firing vs. no firing or brain states under anesthesia which involve cortical up- and down-states.

Within the present study we modified the step-wise strategy for analyzing trajectories by replacing actual activity percolation between brain regions over a time scale of seconds by a different model of spring-like attraction/repulsion between time courses of different brain regions by estimating the respective (directed) spring constants as Kuramoto coupling parameters. By this, we attempt to capture effects of fast neuronal firing on slow fMRI signal. The Kuramoto model has been used in many fields of research to investigate synchronization behavior as it is the most popular and most studied model for this phenomenon. The model can take on many different forms by adding or leaving out parameters. We decided to choose Euler's method for the following reasons: for once, it is the simplest method. Furthermore, we only consider a very short time span (*T*=300) alleviating stability issues, but foremost, we do not use the model for solution generation/simulation but for coefficient estimation. Therefore, the method is appropriate. For future work, however, other methods like the trapezoid method, implicit Euler's method or Runge-Kutta methods could be considered as well. When solving the linear equation system, the solution does not exist a-priori, since the number of equations will in general not be equal to the number of unknown variables. Ill-posedness in the sense that we have less equations than unknown variables will hardly occur, since this would mean that we have less measure points from the recordings than we have IBNs (In our case this meant, that we have <20 measure points, which further meant that our recording was less than about 40 s long). For the over-determined case, however, we need a strategy to find a unique solution that is optimal in a certain sense. The common approach is to solve such problems by finding the best solution in a least-squared error sense. The problem could be proposed in a more general manner, such that different norms than the ℓ_2_-norm are possible. However, interpretability is more difficult in other spaces and distances than Euclidean spaces. Therefore, we solve our problem with the normal equations optimizing the ℓ_2_-norm, although other approaches could also be considered in the future.

We show a correlation of the Ham-D score, assessing clinical disease severity, to *K*, which can very clearly be confirmed in the test data set. The nature of the correlation in exploratory as well as test data set is bipartite with a positive and a negative contribution. Positive correlation indicates that the amplitudes in a set of *K* values increase with increasing disease severity and vice versa. All IBNs included into the analysis contribute to the dependence on Ham-D (except for one in the exploratory data set) indicating a broad change within the inter-regional communication in clinically severe states. In the exploratory data set the directed coupling from the salience network onto the basal ganglia network shows a strong negative correlation to Ham-D. A tendency of a reduced input from the salience network into the basal ganglia matches well with hypotheses of compromised reward processing and anhedonia which is discussed in combination with the dopamine dysregulation hypothesis (Szczypiński and Gola, 2018; Whitton et al., 2020). The correlation of this particular coupling to Ham-D though is not reproduced in the test data set. Our results indicated that clinical severity of MD is associated to a mild change of cross-regional IBN interactions across the whole cortex and sub-cortex.

All our analyses aimed at wide-spread changes. We were able to retrieve global patterns of dependence. The sensitivity of the approach to localized focal changes would need to be addressed with a different appropriate data set.

Using a simulation experiment we provide a proof of principle that our method is able to recover a ground truth, in which a large number of *K*_*i,j*_s depend on an artificial external regressor. Notably, this parametric dependence is not associated to a systematic deviation of the *K*_*i,j*_s from zero. The simulations indicated that increased overall Kuramoto coupling facilitates the re-discovery of the coupling from the data. Conversely, an increasing noise level decreases the ability of our method to recover the ground truth (Supplementary Figure 3). A stronger directional bias in the couplings as well as a strong contrast in coupling dependence on the external regressor between an intrinsically coupled cluster versus the outside of the cluster, also benefit the detection of the parametric dependence. Notably, we only investigated one type of ground truth, although the artificial data simulation leaves a lot of options for design choices. The size of the generated data set was chosen to allow for comparability to the empirical exploratory data set. Also the choices for the eigenfrequencies and the magnitude of the coupling coefficients for the simulation was guided by the empirical data. The magnitude of the coefficients has subordinate impact, as this can—to a certain degree—later be scaled with the appropriate weight *d*.

The most interesting design choices concern the correlation pattern shape **N**. The possibilities here are highly diverse. First, the shape itself can be varied, i.e., which IBNs are involved and in which constellation. Additionally, the number of IBNs within the network, the intensity, pattern, and trend (“gradual progression” vs. “plateau” as for our experiments) of the correlations might be altered. Also the mask matrices used in our synthetic data experiments are only a choice of many more possible variations.

The power of our analysis pipeline for recovering the ground truth of actually parametrically manipulated couplings was limited in the considered simulation settings. Most positives were found in the “boundary” group of couplings, which crossed from one IBN, affected by parametrically dependent coupling to other regions, to another IBN, not affected by any parametrically dependent coupling to other regions. For the analysis of empirical data sets this implies that the reliability of recovering the exact couplings is low, which are actually parametrically dependent on the regressor under concern. More reliable information can be retrieved from the IBNs involved in the set themselves rather than the couplings. We would like to emphasize, that the proposed simulation pipeline and the presented results should be seen as a proof of concept, that it is possible to retrieve parametric dependencies, rather than a validation procedure, since it is impossible to have access to the ground truth of a real world data set. It is important to note, that we did find the induced dependencies in certain scenarios despite the large amount of possible parameter combinations, which supports that we did not find this by chance.

Our approach allows for an estimate of Kuramoto coupling parameters from empirical data and therefore contrasts with other studies which apply generative models in order to simulate and study arising activity dynamics, which are subsequently put in relation to empirical data. This kind of approaches has, e.g., been followed employing the Kuramoto model (Breakspear et al., 2010; Sadilek and Thurner, 2015; Schmidt et al., 2015), the Ising model (Stramaglia et al., 2017), and other spin glass models (Hudetz et al., 2014).

## 6. Conclusion

In summary, we present a novel method for analyzing functional connectivity from fMRI resting state data. Our initial analysis on empirical data indicates that the method provides novel results, which are complementary to other methods established in the field. The focus of the presented analysis pipeline lies on assessing wide spread connectivity changes relating to the brain state and might be useful in the analysis of the relation to slow changing chemical connectivity. The results proved to be robust to a re-test in an entirely independent data set. We further support the validity of our empirical findings by using simulated data, containing a ground truth, in that we show the ability of the method to retrieve this ground truth. Future studies are needed to extend and validate our findings.

## Data Availability Statement

The original contributions presented in the study are publicly available. This data can be found here: https://doi.org/10.6084/m9.figshare.13352399.

## Ethics Statement

The studies involving human participants were reviewed and approved by the in-house Ethics Committee of the Klinikum rechts der Isar, TU Munich for the exploratory data set and by the Investigational Review Board (IRB) at Indiana University School of Medicine and at the Cleveland Clinic Foundation for the test data set. The patients/participants provided their written informed consent to participate in this study.

## Author Contributions

LB: methodology, software, data curation, writing, and visualization. FH, CJ, and MH: validation, investigation, and data curation. PG: supervision. AA: resources and supervision. CP: writing—review and editing and supervision. AW: conceptualization, methodology, software, formal analysis, resources, data curation, writing, and visualization. All authors contributed to the article and approved the submitted version.

## Conflict of Interest

The authors declare that the research was conducted in the absence of any commercial or financial relationships that could be construed as a potential conflict of interest.

## Publisher's Note

All claims expressed in this article are solely those of the authors and do not necessarily represent those of their affiliated organizations, or those of the publisher, the editors and the reviewers. Any product that may be evaluated in this article, or claim that may be made by its manufacturer, is not guaranteed or endorsed by the publisher.

## Footnotes

^1^https://www.mathworks.com/.
