# Supplementary material for: Quantification of Kuramoto Coupling Between Intrinsic Brain Networks Applied to fMRI Data in Major Depressive Disorder
Source: Front Comput Neurosci. 2022 Mar 3;16:729556. doi: 10.3389/fncom.2022.729556 (PMC8929174; doi:10.3389/fncom.2022.729556)
Supplement: Supplementary file 1 [file Data_Sheet_1.pdf]

Appendix A: Validation experiments with artificial data simulation

The general goal of the experiments with synthetically generated data is to gain insight regarding the capabilities of our model. In general, we want to address if induced dependencies on $K_{i,j}$ in model (15) in the simulation process, can be recovered in the generated data with our Kuramoto coupling parameter estimation model described in Sec. 2.2. While we wish to mimic an empirical data set in certain properties, we do not wish to simulate a physiologically accurate signal that represents a biological brain region activity signal, i.e., we do not provide a brain simulation here. The goal is to validate to a certain extend the capabilities of our method. By inducing dependencies on the data generating coefficients, we expect our model to recover these dependencies in the synthetically generated phase data.

The simulation model has already been described in Sec. 4.1. A brief summary of the simulation procedure (Sec. 4.2) and an exemplary result (Sec. 4.3) are also given. In the following sections, we provide details on the simulation and more results from the analysis.

I Simulation pipeline

Fig. 5 shows the steps of the simulation and also highlights points of design choices which are discussed in Sec. 5. We aim to generate a set of phase courses that mimics a real world data set, i.e., a data set of s instances (representing the subjects) with r phase courses (representing the IBN activity phase courses) with T measure points. As input we provide our pipeline with random initial phase values $\varphi_i(0)$, $i = 1, \ldots, r$ and eigenfrequencies ω_i, $i = 1, \ldots, r$ for each subject and each IBN sampled from a Gaussian distribution. For all subjects and all IBNs this can be stored in matrices $\mathbf{\Omega} \in \mathbb{R}^{r \times s}$, $\mathbf{\Phi}_0 \in \mathbb{R}^{r \times s}$. The second input are coupling coefficient matrices $\mathbf{K}^{\mathrm{o}}$ of size $r \times r$ for each subject, which are also generated with random values. The third input is an independent *score* vector $\mathbf{s}$ for the s subjects (representing the regressor Ham-D score). As a next step certain coefficients are altered for all subjects such that they are correlated with $\mathbf{s}$ across subjects. The coefficients to be altered are chosen such that they build what we call a correlation pattern $\mathbf{N}$. Specific connected coupling coefficients show a high correlation across subjects while the remaining coefficients show weaker correlations. The forced correlations are achieved by the procedure depicted in the top white box of the second column (parametric dependence) in Fig. 5. Plotting a coefficient against $\mathbf{s}$ across subjects will more or less randomly distribute the points on the plane for the randomly initialized $\mathbf{K}^{\mathrm{o}}$ matrices (dark red dots). A straight line is generated in the plane and each point is altered such that it is pushed closer to this line, thereby increasing correlation (light blue points). Note, that this procedure can force positive and negative correlation to a desired degree by drawing the straight line with positive or negative slope, respectively and by weaker or stronger pushing of the points to the line. The altered coefficient matrices are termed $\mathbf{K}^{\mathrm{c}}$. In Fig. 6a the correlation coefficients of each coupling coefficient with $\mathbf{s}$ are plotted in ascending order, before (dark red circles, $K^{\mathrm{o}}_{i,j}$) and after (blue circles, $K^{\mathrm{c}}_{i,j}$) a manipulation. Note, that this implies that now the $K_{i,j}$ coefficient number is not equal to the index of the circle in the figure (thus, e.g., the first plotted circle does not necessarily represent the correlation of $K_{1,2}$ with $\mathbf{s}$). As can be seen in the top right corner, quite a few coefficients are highly correlated with $\mathbf{s}$ across subjects after the manipulation, while before, the range of correlation was basically limited to the interval $[-0.6, 0.6]$. We further generate mask matrices $\mathbf{M}$ of the same size as $\mathbf{K}$ which additionally allow to select a certain portion of the coefficients or weight the influence of, e.g, coefficients with high correlations higher than those with weak correlations. Additionally, a general weight parameter d is included acting equally on all coefficients (in contrast to $\mathbf{M}$). Gaussian noise is also added to the data, weighted with n. For the phase course generation, we now have all parameters to insert them into our Kuramoto model (see Sec. 4.1). From the initial phase values and the eigenfrequencies the model iteratively calculates further values. For the resulting $s \times r \times T$ phase course data set for a certain mask $\mathbf{M}$, correlation pattern $\mathbf{N}$, d and n combination the $\mathbf{K}^{\mathrm{res}}$ coefficients are then calculated with our model (see Sec. 2.2) for each subject. These (re-)calculated Kuramoto coefficients $\mathbb{K}^{\mathrm{res}}$ are then analyzed with the same statistical cluster permutation test that was used for the empirical data sets. The hypothesis here is that if we systematically induce correlations of the simulation guiding coupling coefficients $\mathbb{K}^{\mathrm{c}}$ on the regressor across subjects, we expect to detect systematic correlations in the recalculated $\mathbb{K}^{\mathrm{res}}$ coefficients again.

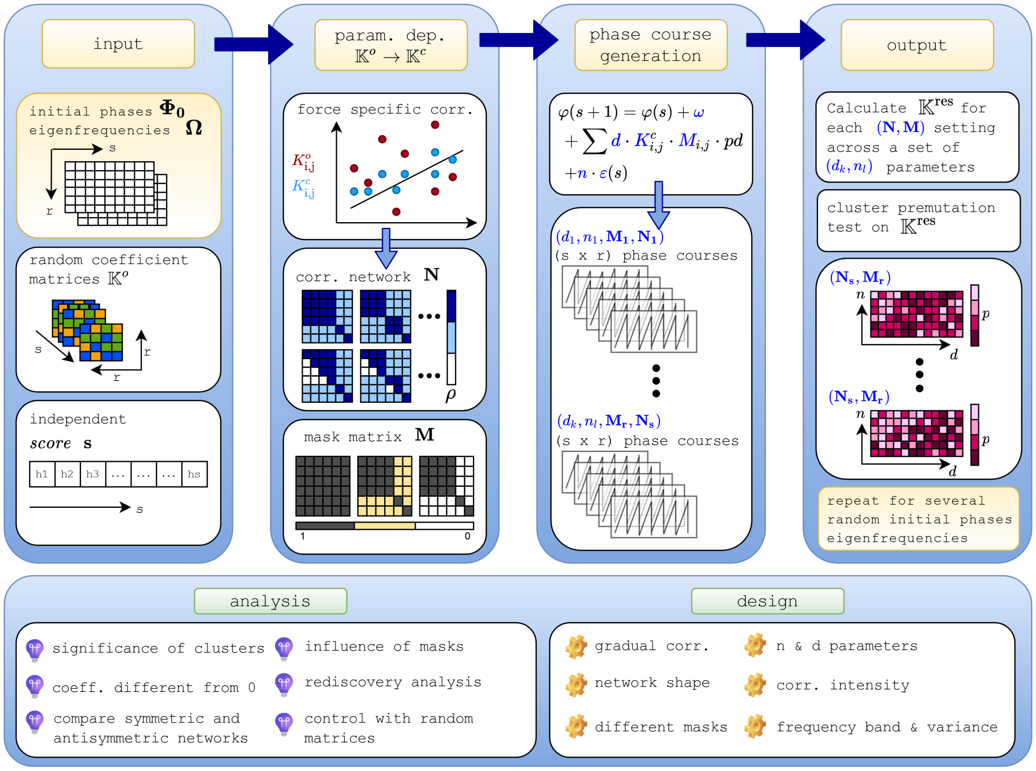

Figure 5: **Artificial data analysis pipeline**. **Input.** We provide our pipeline with random initial phase values and eigenfrequencies for each subject and each IBN. The second input are coupling coefficient matrices $\mathbb{K}^{\text{o}}$ for each subject generated with random values. The third input is an independent *score* vector s for the subjects. **Insertion of parametric dependence on $\mathbb{K}^{\text{o}}$.** As a next step certain coefficients are altered such that they are correlated with the inserted score across subjects. The coefficients to be altered are chosen, such that they build a correlation pattern **N**. We further generate mask matrices **M** of the same size which additionally allow to weight certain coefficients differently in the following step. **Phase course generation.** We use the Kuramoto model to generate the $s \times r \times T$ phase course data set inserting the mask matrix **M** and the altered coefficient matrices $\mathbb{K}^{\text{c}}$ (pd = phase differences). Additionally, a general damping parameter d, acting equally on all coefficients and Gaussian noise weight with n is included in the process. For each mask $\mathbf{M}_r$ and pattern $\mathbf{N}_s$ scenario, the $s \times r \times T$ phase courses are generated for a set of different overall coupling strength d and noise level n values. **Output.** For the resulting data set the Kuramoto coefficients are calculated again $\mathbb{K}^{\text{res}}$ and analyzed with the same statistical cluster permutation test as the empirical data sets. **Analysis and Design.** The bottom box summarizes points of interest such as if we are able to retrieve induced correlations in $\mathbb{K}^{\text{c}}$ in the recalculated coefficients $\mathbb{K}^{\text{res}}$. The design bullets list several design choices of the simulation process emphasizing the range of possible future investigation directions.

i) Parameter choices

Technical parameters: Since the model for the generation of the synthetic data set includes several parameters, their specific choice naturally influences the results. Some parameters, which we summarize as *Technical Parameters*, are simply chosen such that we, in general, best mimic the empirical data set to provide maximum comparability. This includes, for instance, the size of our data set ($s \times r \times T = 24 \times 20 \times 300$). Details about the specific choices for these parameters can be found in the Supplement (see Sec. 3.2).

To maintain a certain balance between synchronization and de-synchronization, the weight factor d needed to be set such that the oscillators deviate from their respective eigenfrequencies, but at the same time the influence needs to be damped to prevent highly noisy and strongly oscillating phase courses. Starting above a d value of 0.6, coupling leads to severe impact on the phase time courses, to an extent that the overall spectral properties of the time courses are distorted. As this does not reflect

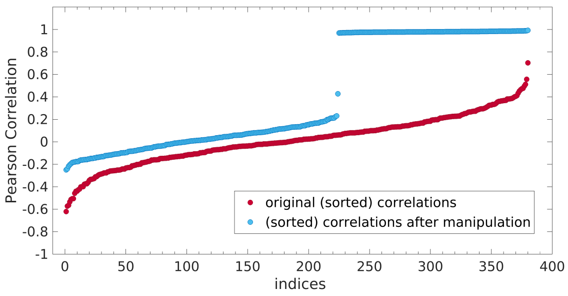

(a) Correlations in ascending order for the random initial coefficients and after explicit insertion of (symmetric) parametric dependence.

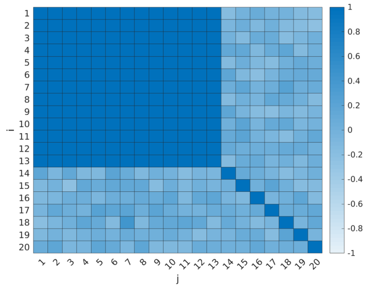

(b) Correlation pattern $\mathbf{N}_{\text{s}}$.

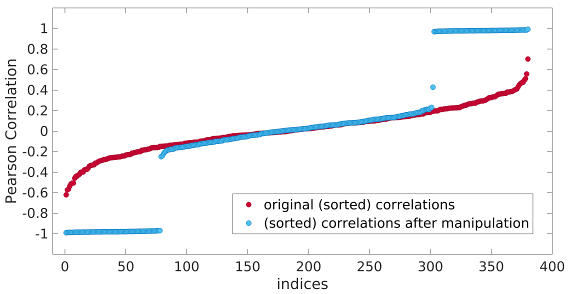

(c) Correlations in ascending order for the random initial coefficients and after explicit insertion of (asymmetric) parametric dependence.

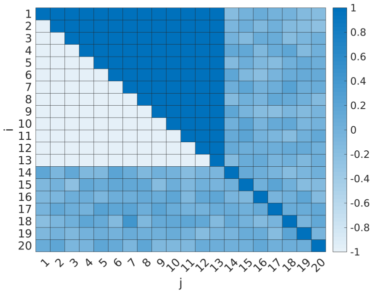

(d) Correlation pattern $\mathbf{N}_{\text{as}}$.

Figure 6: **Correlation patterns.** We manipulated specific coefficients to create a 'network of IBNs' whose pair-wise coupling coefficients significantly correlate with the score $\mathbf{s}$. **(a)** Each circle in dark red shows the correlation coefficient of one $K^{\text{c}}_{i,j}$ coefficient with the score $\mathbf{s}$ based on the randomly generated $\mathbb{K}^{\text{o}}$-matrices. The blue circles show the same but for the matrices where a certain portion of the coefficients is manipulated. **(b)** Plotting a heat map of the correlations of all coefficients against $\mathbf{s}$ yields the pattern shown in the top right plot. This pattern is termed $\mathbf{N}_{\text{s}}$. **(c) and (d)** We manipulate the same coefficients but the correlation pattern is asymmetric in the sense that if $K^{\text{c}}_{i,j}$ is highly positive correlated, $K^{\text{c}}_{j,i}$ is necessarily highly negative correlated to $\mathbf{s}$. This pattern is termed $\mathbf{N}_{\text{as}}$.

a realistic scenario we limited our simulations to d values within the range of $[0.01, 0.6]$. An example of a typical phase course generated by our simulation is shown in Fig. 5 in the Supplement. Gaussian noise was added with intensity levels within the range $[0.5, 3]$ resulting in 5% to 30% noise, which falls into the regime of stochastic resonance in modelling of fMRI time courses (Deco et al., 2009).

The choices of the remaining parameters, i.e., the correlation pattern $\mathbf{N}$ and mask matrices $\mathbf{M}$, is guided by specific research questions. The overall main question is: If we induce certain dependencies of the simulation guiding coupling coefficients $\mathbb{K}^{\text{c}}$ on the independent score $\mathbf{s}$ across subjects, can we recover these dependencies in the generated data with our Kuramoto coupling estimation model and if so under which circumstances and to what degree?

Correlation pattern $\mathbf{N}$ and mask matrices $\mathbf{M}$: We manipulated a certain portion of coefficients to construct the correlation patterns depicted in Fig. 6. As can be seen in Fig. 6b and 6d, regions 1 to 13 are coupled with coefficients that show significant correlations, while the other regions are considered 'outside the correlation network'. In Fig. 6b all significant correlations are highly positive yielding a symmetric correlation pattern. We will term this correlation pattern $\mathbf{N}_{\text{s}}$. In Fig. 6d correlations 'within the correlation network' show an asymmetric correlation pattern, i.e., if $K^{\text{c}}_{i,j}$ is highly positively correlated, $K^{\text{c}}_{j,i}$ is necessarily highly negatively correlated to $\mathbf{s}$. This pattern is termed $\mathbf{N}_{\text{as}}$.

Furthermore, we consider three cases of mask matrices $\mathbf{M}$, which will be multiplied point-wise with the coefficient matrix $\mathbf{K}^{\text{c}}$ for each subject. This allows to weight a certain portion of coefficients differently or even exclude certain coefficients in the simulation process by setting the respective $\mathbf{M}$ entry to zero. Fig. 7 shows in the left column the three masks we used in our experiments.

For a fixed parameter setting ($\mathbb{K}^{c} \in \mathbb{R}^{24\times20\times20}, \mathbf{\Omega} \in \mathbb{R}^{20\times24}, \mathbf{\Phi_0} \in \mathbb{R}^{20\times24}, \mathbf{N}, \mathbf{M} \in \mathbb{R}^{20\times20}, d \in \mathbb{R}^{+}, n \in \mathbb{R}^{+}$), the $24 \times 20 \times 300$ phase course data were calculated and subsequently, a Kuramoto coupling coefficient matrix is calculated for each subject with our Kuramoto coupling estimation model, which results in 24 matrices of size 20×20 ($\mathbb{K}^{\text{res}}$). To account for the randomness in the initialization of $\mathbf{\Omega}$ and $\mathbf{\Phi_0}$, we repeat the simulation pipeline for six different random phase and eigenvalue initializations and perform evaluations in account of these six runs, i.e., averages and medians, depending on the evaluation metric at hand. We equidistantly set d and n within their limited value ranges and made calculations with all combinations of theses two parameters while keeping the other parameters fixed. The simulation process is quite run time intensive, therefore, we choose the number of runs to stay within a moderate run time but at the same time account to a certain degree for the randomness of the initialization. For the cluster permutations test on the resulting $\mathbb{K}^{\text{res}}$, bi-directional correlations were considered with a number of 100 permutations.

II Results on simulated data

By employing the Kuramoto model to fMRI data we were decidedly looking for mild wide spread alterations in coupling strength, and hypothesized these would be impacting on the spectrum of brain processes. This was supported by our detection of an actual dependence of Ham-D on the detected K parameters without them differing significantly in amplitude towards zero or between groups. While this closely matches our initial hypothesis, it is rather complementary to findings from other measures, showing significant deviations of amplitudes from zero, but no parametric dependence on Ham-D. Therefore, with the artificial data simulations, we were aiming to confirm that our procedures actually allow for retrieving parametric dependencies of K parameters to an external regressor.

i) Significance on the set level with cluster permutation test depending on noise level n and overall coupling weight d

We found that parametric dependencies of Kuramoto coupling on an external regressor in subsets of couplings can be recovered by our artificial data analysis pipeline (see Fig. 7). In the following, we will describe the results in more detail.

ii) Dependence on correlation pattern $\mathbf{N}$ and on mask matrices $\mathbf{M}$.

We use simulations from two models on how a subset of Kuramoto coupling parameters would depend on the artificial regressor $\mathbf{s}$, namely the two correlation patterns $\mathbf{N}_{\text{s}}$ and $\mathbf{N}_{\text{as}}$. Furthermore, we used the three mask matrices, weighting coefficients 'within the correlation network' differently than those 'outside the correlation network'. Fig. 7 shows the cluster permutation test median P-values over the six runs for $\mathbf{N}_{\text{s}}$ (middle column) and the three mask matrices. The right column shows the same for $\mathbf{N}_{\text{as}}$. The simulations on the two studied examples show that couplings between two IBNs with a strong directional bias (Fig. 7, right column) can be recovered more easily by the method. In the case in which parametric dependence operated in the same direction, i.e. positive dependence on the score $\mathbf{s}$ (Fig. 7, middle column), the parametric dependence could only be recovered for higher impacts of coupling, d. Furthermore, a higher contrast in dependence of the $\mathbb{K}^{c}$ parameters outside the set of coefficients with significant correlations to within this set leads to easier recovering of dependencies in both cases of $\mathbf{N}$ (Fig. 7a vs Fig. 7b and Fig. 7c). In Fig. 10 and 11 of the Supplement (see Sec. 5.1), we present more results for three additional mask matrices ($\mathbf{M}_{0.5/0}$, $\mathbf{M}_{1/0.5}$ and $\mathbf{M}_{1/0.25}$). Results follow the same pattern as observed for the scenarios described in Fig. 7.

iii) Parameters determined by the analysis pipeline on the choice of artificial data deviated from zero to a very limited extent

For all runs of all parameter settings we applied a Wilcoxon signed rank test for all coefficients $K^{\text{res}}_{i,j}$ (Bonferroni corrected for multiple testing with 380), if coefficients were significantly different from zero. There was no substantial evidence for the detected coefficients to significantly deviate from zero. Single coefficients in single runs did result in a positive test rejecting the $\mathcal{H}_0$ but it was never the same coefficient in the same (n, d) setting across runs and significant coefficients were not systematically present in significant clusters. We did not correct for multiple testing neither for the 174 (n, d) settings nor the six runs, since in real world settings, we would not have these different

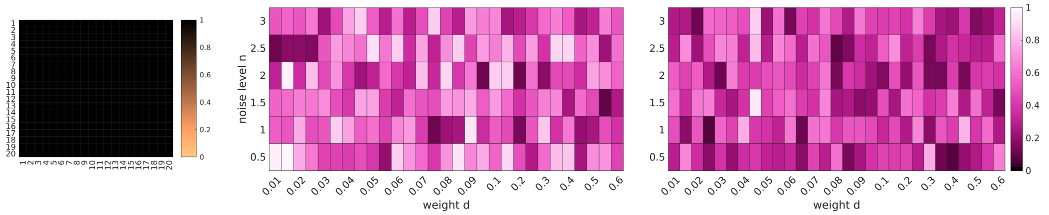

(a) $\mathbf{M}_{1/1}$ (left) and median P-values for $\mathbf{N}_\text{s}$ (middle) and $\mathbf{N}_\text{as}$ (right).

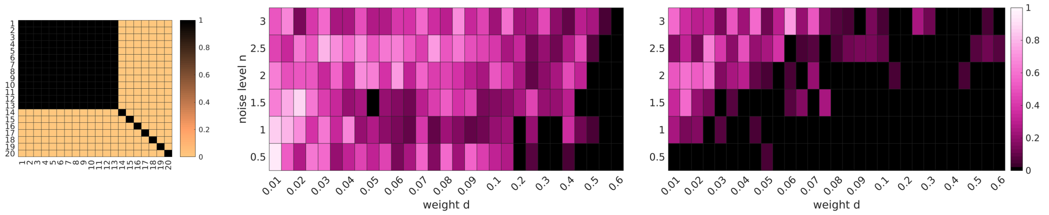

(b) $\mathbf{M}_{1/0}$ (left) and median P-values for $\mathbf{N}_\text{s}$ (middle) and $\mathbf{N}_\text{as}$ (right).

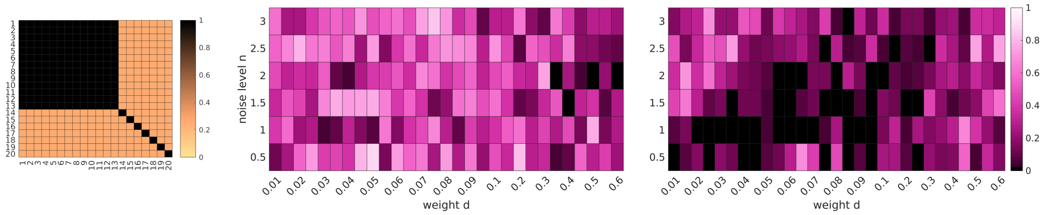

(c) $\mathbf{M}_{1/0.1}$ (left) and median P-values for $\mathbf{N}_\text{s}$ (middle) and $\mathbf{N}_\text{as}$ (right).

Figure 7: **Artificial data simulation cluster permutation test results.** Each subplot in the middle and right column shows the median P-values over the six runs for all (n, d) combinations. The middle column shows results for $\mathbb{K}^\text{c}$ with the correlation pattern $\mathbf{N}_\text{s}$, the right column shows results for $\mathbb{K}^\text{c}$ with the correlation pattern $\mathbf{N}_\text{as}$. Cells with significant P-values (< 0.05) are colored in black. In each subplot (a), (b), and (c) another mask matrix was used. The respective mask matrix is shown to the left of the two corresponding median P-value result plots (left column). The sub captions contain their naming, where the subscripts in $\mathbf{M}_\text{in/out}$ should be interpreted as follows: The number at the position 'in' is the weight for the coefficients with distinct correlations ('inside the correlation structures' $\mathbf{N}_\text{s}$ and $\mathbf{N}_\text{as}$ respectively), while the number in the subscript position 'out' is the weight for all coefficients with a very weak correlation ('outside these correlation structures'). For example, in the case of (c) $M_{1/0.1}$, the coefficients with induced strong positive or negative correlations are 10 times as active as other coefficients. Comparing the two correlation structures, we can see, that retrieving significant clusters was easier when the correlation pattern was asymmetric (right vs middle column). Increasing d leads to a better capacity of the analysis pipeline to retrieve the significant dependence on the score s from the artificial data. Added noise ε in the coupling process has a detrimental effect on recovering the dependence on s. Considering the different mask matrices, we can observe, that a strong contrast in dependence of the $\mathbb{K}^\text{c}$ parameters, i.e., coefficients outside the closed cluster are set to zero (b), or have very weak influence (c) leads to more prominent significance recovery, especially for the asymmetric correlation pattern $\mathbf{N}_\text{as}$.

cases. However, multiple tests were executed in our artificial data setting and an additional correction with the factor 6 accounting for multiple runs would lead to a single coefficient with a positive test result. It was, however, a coefficient, where the (n, d) setting did not result in a significant cluster and the coefficient was additionally not even in the cluster. Table 2 in the Supplement Sec. 5.2 summarizes the results for all mask matrices and both correlation patterns.

iv) Control simulations with initial random matrices indicate a larger impact of correlations than masks.

To see the effect of the correlation manipulation, we repeated the same analysis pipeline as for $\mathbb{K}^{c}$, for the original random $\mathbb{K}^{o}$ matrices, whose correlations correspond to the dark red circles in Fig. 6a (Fig. 6c, respectively). If we simulate phase courses where the coefficients do not show a significant correlation with the the score $\mathbf{s}$ (i.e., $\mathbb{K}^{o}$ and mask $\mathbf{M}_{1/1}$), the significance in the statistical analysis shows a random behavior. Some parameter combinations indicate the finding of a network cluster associated with $\mathbf{s}$, but this is not systematically related to the intensity of the coefficients d in the simulation or the noise level n. In general, fewer and less significant results are found. Since this is also the case for $\mathbf{N}_{s}$ and $\mathbf{N}_{as}$, we investigated the results when applying the mask $\mathbf{M}_{1/0}$, since this led to systematically significant network clusters. Again the results show a random significance pattern, which indicates a strong impact on the results from the correlations and a weaker impact of the exclusion of certain coefficients by setting them to zero. The results can be seen in Fig. 12 in the Supplement (see Sec. 5.3). We conclude that the results for finding significant clusters is indeed largely impacted by the correlations of coefficients and score $\mathbf{s}$ and cannot be retrieved by solely excluding certain coefficients.

v) Rediscovery analysis

We analyzed in how far the exact couplings parametrically depending on the score $\mathbf{s}$ could be recovered by the analysis pipeline. We, therefore, separated couplings into three categories being the couplings actually parametrically manipulated ('inside correlation pattern coefficients', IC), those couplings between an IBN from within the closed correlation pattern and an 'outside' IBN ('bridging couplings', BC), and those only connecting IBNs from outside the set of manipulated coefficients ('reference couplings', RC). In this last group of reference couplings the detection of a significant parametric dependence of the $\mathbb{K}^{res}$ parameters on the score $\mathbf{s}$ was regarded as false positive. In the group of 'bridging couplings' such a dependence would highlight at least one region actually affected by true parametric dependence on $\mathbf{s}$. We found that in general, couplings of group BC had the highest likelihood of being included into a significant set. We conclude, the reliability of significant coefficients can be mainly addressed on an IBN level, but not necessarily for the specific coefficient. In the Supplement the analysis design and the results are described in detail (see Sec. 3.3 and 5.4 in the Supplement).
