# Supplementary material for: Quantification of Kuramoto Coupling Between Intrinsic Brain Networks Applied to fMRI Data in Major Depressive Disorder
Source: Front Comput Neurosci. 2022 Mar 3;16:729556. doi: 10.3389/fncom.2022.729556 (PMC8929174; doi:10.3389/fncom.2022.729556)
Supplement: Supplementary file 2 [file Data_Sheet_2.pdf]

Supplementary Material to: Quantification of Kuramoto coupling between intrinsic brain networks applied to fMRI data in major depressive disorder

In the following sections we include additional information, results, figures and derivations that did not fit the scope of the main paper.

1 Data

1.1 Demographic data of empirical data sets

Table 1 summarizes demographic data of the empirical data which are described in detail in Sec. 2.1.1 in the main paper.

	Exploratory Data Set			Test Data Set		
	HC mean (SD)	MD mean (SD)	MD vs. HC P	HC mean (SD)	MD mean (SD)	MD vs. HC P
N	24	24		37	44	
age / y (mean [min/max])	44 [26/68]	48 [23/79]	$> 0.05^a$	24 [18/30])	24 [18/30]	$> 0.05^a$
sex (m/f)	10/14	11/13	$> 0.05^b$	15/22	11/33	$> 0.05^b$
Clinical parameters						
duration of current episode / weeks	NA	16 (7)		NA	28 (55)	
number of depressive episodes	NA	6 (3)		NA	23 (32)	
medication free period / weeks	NA	NA		NA	91 (126)	
number of medication naïve patients	NA	NA		NA	17	
Ham-D	0	21.8 (7.1)	$< 0.001^{c,*}$	0.4 (0.7)	16.5 (3.3)	$< 0.001^{c,*}$
YMRS	–	none^d		0	0.11 (0.32)	$= 0.036^{c,*}$
Movement parameters						
volume-wise mean motion (mm)	0.94 (0.44)	1.21 (0.79)	$> 0.05^a$	0.36 (0.12)	0.36 (0.11)	$> 0.05^a$
motion correlation to SC_{VTA}, P	$> 0.05^e$	$> 0.05^e$		$> 0.05^e$	$> 0.05^e$	
motion correlation to SC_{DRN}, P	$> 0.05^e$	$> 0.05^e$		$> 0.05^e$	$> 0.05^e$	
motion correlation to Ham-D, P	NA	$0.034^{e,*}$		NA	$> 0.05^e$	

Table 1: **Demographic and clinical data.** ^{a}two-sample t-test; $^b\chi^2$-test; ^{c}Wilcoxon rank sum test; ^{d}no symptoms of mania in clinical interview; ^{e}Spearman correlation; *significant for p < 0.05; Abbreviations: HC, healthy controls; MD, major depressive disorder; SD, standard deviation; NA, not applicable; Ham-D, Hamilton Depression Rating Scale; YMRS, Young Mania Rating Scale

The relevance of the dependence of Ham-D on head movement is discussed in section 4.1 of the Supplement.

1.2 IBN layouts

Fig. 1 shows the 20 IBNs detected in the empirical data set. Fig. 2 shows the 28 IBNs detected in the test data set.

1.3 Metastability Analysis

In order to measure metastability, the averaged synchronization of extracted IBNs was measured using the Kuramoto order parameter R (Deco et al., 2017). All IBN time courses were band pass filtered to 6 frequency bins using a 7th order Butterworth filter: 0.01 Hz - 0.025 Hz, 0.025 Hz - 0.05 Hz, 0.05 Hz - 0.075 Hz, 0.075 Hz - 0.1 Hz, 0.1 Hz - 0.125 Hz. $R(t)$ across all filtered IBN time courses was calculated per band. $R(t)$ measures global level of synchronization of n oscillating

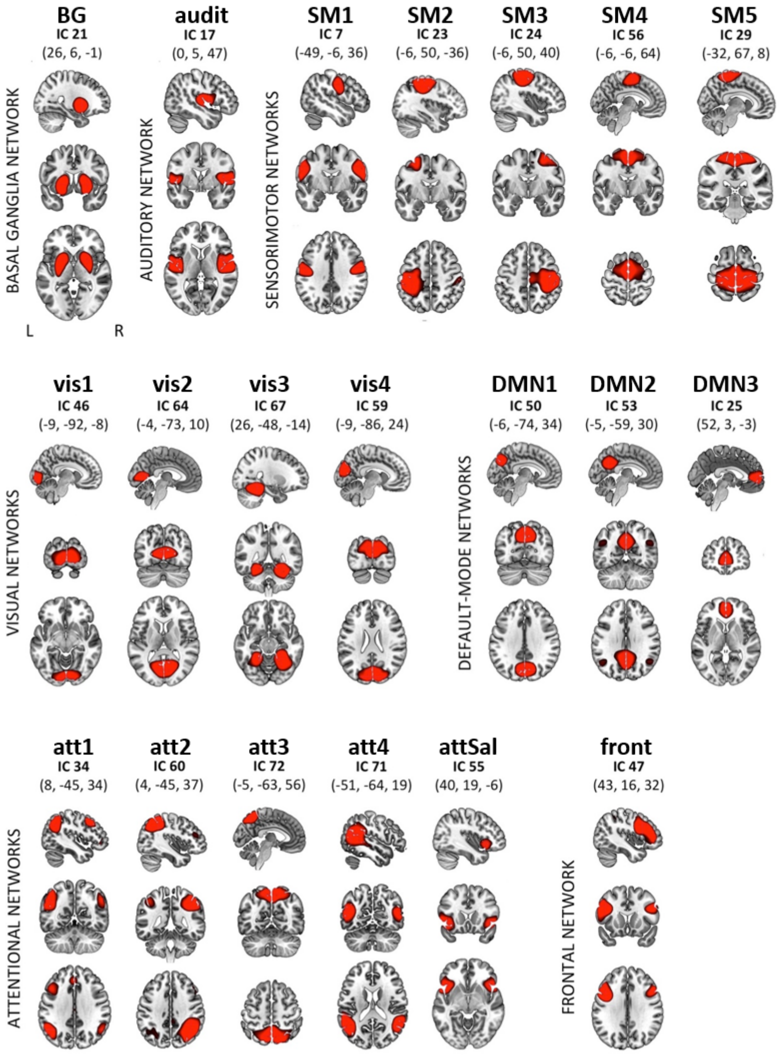

Figure 1: IBN layouts of the 20 independent components (ICs) identified in the exploratory data set, obtained from spatial group-ICA, plotted as z-scores, thresholded at z>1, and displayed at the three most informative slices (MNI space). Networks are categorized into groups according to anatomical and functional properties. IC numbers refer to the matching networks in Allen et al., 2011.

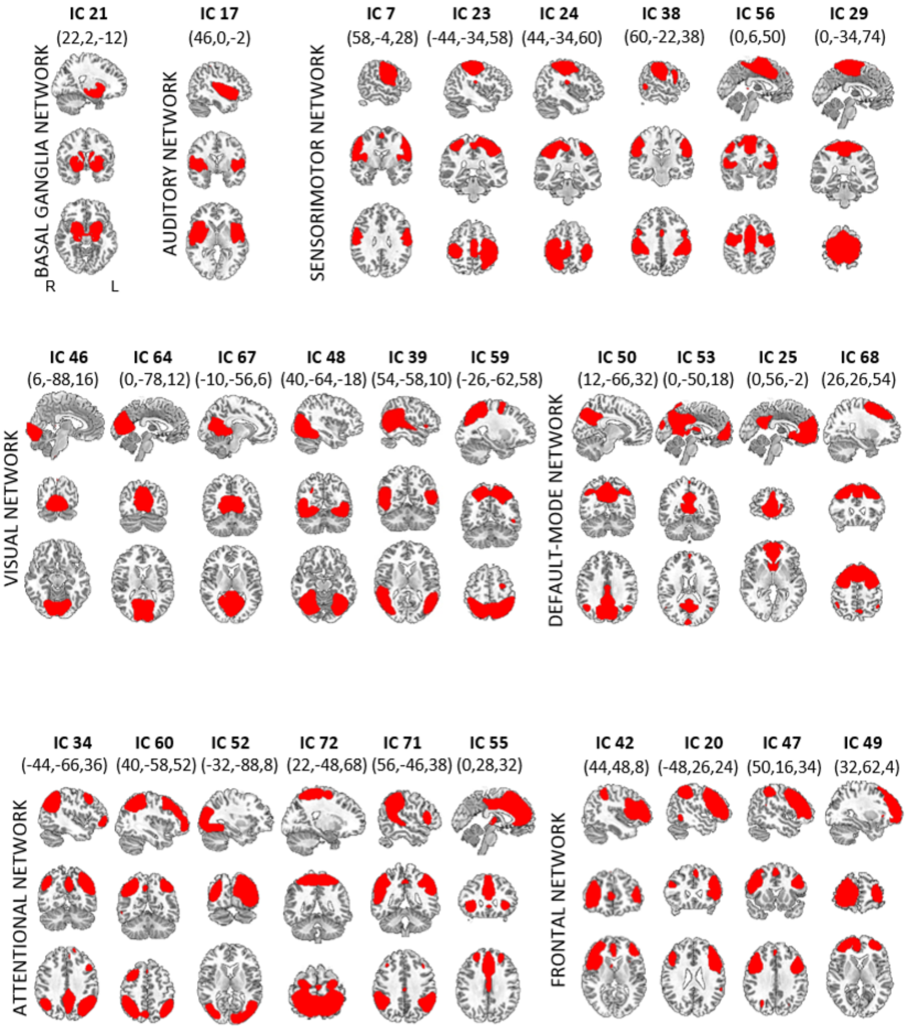

Figure 2: IBN layouts of the 28 independent components (ICs) identified in the test data set, obtained from spatial group-ICA, plotted as z-scores, thresholded at z>1, and displayed at the three most informative slices (MNI space). Networks are categorized into groups according to anatomical and functional properties. IC numbers refer to the matching networks in Allen et al., 2011.

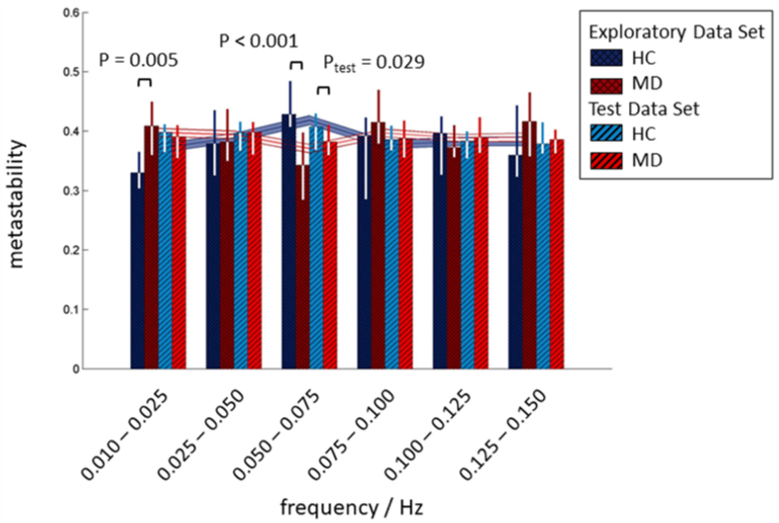

Figure 3: Median metastability measures per participant group and frequency bin with 50% confidence intervals. Additionally, the overall means and standard deviations across all patients and all controls, respectively, are indicated as line plot. Relevant significant figures marked. Analysis on the exploratory data set revealed a significant metastability reduction at frequencies 0.05 Hz to 0.075 Hz, which was tested and confirmed in the test data set.

signals. When a lack of synchronization is present the n phases are randomly distributed equating to R valued at near 0. If all n oscillating signals are synchronized, this will result in $R = 1$ (full synchronization). The mathematical expression of the Kuramoto order parameter is given by:

$$R(t) = \frac{1}{n}\Big|\sum_{k=1}^{n} e^{i\phi_k(t)}\Big|, \tag{1}$$

where $\phi_k(t)$ is the instantaneous phase of each narrow-band BOLD signal at node k. The instantaneous phase $\phi_k(t)$ of each narrowband signal was computed using the Hilbert transform (as implemented in matlab, www.mathworks.com). Values of $R(t)$ were streched from the range of 0 to 1 to the range of -1 to 1 and Fisher Z transformed to the full range of real numbers in order the measure the standard deviation across time. In taking the standard deviation across time, the number of transitions from synchronized to desynchronized states can be quantified, representing metastability.

Statistical analysis was first performed on the exploratory data set. Metastability values per bin were tested for normality by a Lilliefors test implemented in matlab (www.mathworks.com). Distributions proved not to be normal. Therefore, statistical group comparisons between patients and controls per each of the nine frequency bins were performed as Wilcoxon rank-sum tests implemented in matlab. Here, group differences were regarded as significant with at $P_c < 0.05$, Bonferroni corrected for the 6 tests resulting from the splitting into 6 frequency bands. The detected significant group differences provided the a very specific a priori hypothesis for the independent test data set regarding a metastability reduction in frequencies of 0.05 Hz to 0.075 Hz. Based on this, the group difference in the test sample was tested for significance by a right-tailed Wilcoxon rank-sum testing without Bonferroni correction for number of frequency bins and proofed to be significant (see Fig. 3).

In consequence we performed our analysis of K in the frequency bin of 0.05 Hz to 0.075 Hz.

2 Calculation of Kuramoto coupling parameters K

2.1 Kuramoto model

Synchronization denotes the phenomenon of several processes with individual rhythms coming into co-occurrence with a common rhythm after interacting dynamically. This effect has been observed and described in many different fields of research such as sociology, biology, technology and ecology (Arenas et al., 2008). The first attempt to describe synchronization in mathematical terms was done in

1967 by Winfree (Winfree, 1967) as he viewed the interacting units as phase oscillators. Since then, many synchronization-based models have been established of which the Kuramoto model (Kuramoto, 1975) is one of the most widely investigated (Acebrón et al., 2005; Bocharova, 2014). In the classical so called *mean field coupling* form it can be written as

$$\dot{\varphi}_i(t) = \frac{\partial \varphi_i(t)}{\partial t} = \omega_i + \frac{C}{r}\sum_{j=1}^{r} \sin(\varphi_j(t) - \varphi_i(t)) = f(t, \varphi_i(t)) \quad i = 1, \ldots, r, \tag{2}$$

where the interaction between oscillators $\varphi_i(t)$, $i = 1, \ldots, r$ is determined by the constant coupling strength C. We allow individual coupling by replacing C by coefficients $K_{i,j}$. Furthermore, model (2) incorporates that each oscillator rotates at its own natural frequencies ω_i which is taken from a distribution $g(\omega)$. While the coupling strength forces the frequencies to assimilate, the natural frequencies work as the opposite force. The larger the variance of $g(\omega)$, the less likely will the phases of the oscillators synchronize. The time courses in our experiments are filtered to a very narrow frequency band. Therefore, we chose to model the eigen-frequencies as the mean frequency of the respective frequency band. Another option would be to estimate each eigen-frequency from the largest peak in the frequency profile of each time course via fast Fourier transform (FFT). Our codes provide both options.

2.2 Euler's Method

Given the ODE

$$\dot{\varphi}(t) = f(t, \varphi(t))$$

Euler's method (Epperson, 2013) for a step-wise approximation of the solution reads as follows

$$\hat{\varphi}(t + h) = \hat{\varphi}(t) + h \cdot f(t, \hat{\varphi}(t)),$$

where h is a fixed step size. The smaller h, the more accurate is the approximation $\hat{\varphi}$ to the real solution φ. If we tried to solve our system of ODEs with this method, we would have to address stability questions, as for stiff ODEs, the Euler method is only stable for extremely small step sizes. In this work, however, we start with the exact values of the time courses and calculate the coefficients of the equation. Therefore, in the end, the step size h will only affect the calculations as a scaling factor for the coefficients $K_{i,j}$, which will become apparent, when we rearrange the equations to the LES (see Sec. 2.3). Furthermore, we are only considering a very short time span, and only a relatively small number of steps, which alleviates concerns about stability. In future work investigating with other methods such as Runge-Kutta procedures would of course be possible as well.

2.3 Derivation of the LES

We derive the linear equation system (LES) matrix and inhomogeneity, starting from the following equation, which was derived by plugging in the phase values into Euler's method. As a first step, we bring $\varphi_i(s)$ and ω_i to the left side of the equation and multiply the whole expression by 20. The factor 20 could be omitted, since it will only lead to an equal scaling of the coefficients. Nevertheless, we included it in our calculations to account for the 20 brain regions.

$$\varphi_i(s+1) = \varphi_i(s) + \omega_i + \frac{1}{20}\sum_{j=1}^{20} K_{i,j} \sin(\varphi_j(s) - \varphi_i(s)) \qquad | -\varphi_i(s) \mid \cdot 20$$

$$20 \cdot (\varphi_i(s+1) - \varphi_i(s) - \omega_i) = \sum_{j=1}^{20} K_{i,j} \sin(\varphi_j(s) - \varphi_i(s))$$

We then rewrite the sum on the right hand side as a vector-vector multiplication.

$$\underbrace{20 \cdot (\varphi_i(s+1) - \varphi_i(s) - \omega_i)}_{\text{one entry of } \mathbf{b}_i} = \underbrace{\begin{bmatrix}\sin(\varphi_1(s) - \varphi_i(s)) & \cdots & \sin(\varphi_{20}(s) - \varphi_i(s))\end{bmatrix}}_{\text{one row of the LES coefficient matrix } \mathbf{S}_i} \cdot \underbrace{\begin{bmatrix} K_{i,1} \\ \vdots \\ K_{i,20} \end{bmatrix}}_{\text{vector of unknown coefficients}}$$

For each $s = 0, \ldots, 298$ we obtain one entry for $\mathbf{b}_i$ and one corresponding row for $\mathbf{S}_i$. This leads to the following matrix-vector form:

$$\mathbf{S}_i = \begin{pmatrix} \sin(\varphi_1(0) - \varphi_i(0)) & \sin(\varphi_2(0) - \varphi_i(0)) & \cdots & \sin(\varphi_{20}(0) - \varphi_i(0)) \\ \sin(\varphi_1(1) - \varphi_i(1)) & \sin(\varphi_2(1) - \varphi_i(1)) & \cdots & \sin(\varphi_{20}(1) - \varphi_i(1)) \\ \vdots & \vdots & \ddots & \vdots \\ \sin(\varphi_1(298) - \varphi_i(298)) & \sin(\varphi_2(298) - \varphi_i(298)) & \cdots & \sin(\varphi_{20}(298) - \varphi_i(298)) \end{pmatrix}$$

$$\mathbf{K}_i = \begin{pmatrix} K_{i,1} \\ K_{i,2} \\ \vdots \\ K_{i,20} \end{pmatrix} \qquad \mathbf{b}_i = 20 \cdot \begin{pmatrix} \varphi_i(1) - \varphi_i(0) - \omega_i \\ \varphi_i(2) - \varphi_i(1) - \omega_i \\ \vdots \\ \varphi_i(299) - \varphi_i(298) - \omega_i \end{pmatrix}$$

Note, that the matrix $\mathbf{S}_i$ does not contain the entry for $t = 299$. This is due to the fact, that we only obtain 299 entries for $\mathbf{b}_i$, since it contains differences of consecutive φ_i values. We have to omit the last entry in order to have matching dimensions of the system matrix and the inhomogeneity vector. Here, we also see, that the matrix $\mathbf{S}_i$ will contain a zero column. To be precise, the i-th column will only contain zero values, as the sine of zero is zero. This results in a singular matrix, for which we cannot calculate a solution vector. Therefore, we have to eliminate this column and the corresponding unknown variable $K_{i,i}$, which we simply set to a fixed value of 1.

2.4 Optimized Solution - Gaussian Normal Equations

In order to obtain the coefficients $K_{i,j}$, $i, j = 1, \ldots, 20$, $i \neq j$ we have to solve an over-determined LES, which can be written in a general form as:

$$\mathbf{S} \cdot \mathbf{x} = \mathbf{b}, \tag{3}$$

where the system matrix $\mathbf{S} \in \mathbb{R}^{n \times m}$ with $n \geq m$, the solution vector $\mathbf{x} \in \mathbb{R}^{m \times 1}$ and the inhomogeneity $\mathbf{b} \in \mathbb{R}^{n \times 1}$. This means we have more equations than unknowns. We proposed to accept an approximated solution $\hat{\mathbf{x}}$ for which we obtain the least squared error. Thus, if we define

$$e_k = \sum_{l=1}^{m} S_{k,l} \cdot x_l - b_k \quad k = 1, \ldots, n$$

the error of $\mathbf{x}$ in the k-th row, or equation respectively, we want $\mathbf{x}$ to be chosen such that

$$E(\mathbf{x}) = \sum_{k=1}^{n} e_k^2 = \mathbf{e}^T \cdot \mathbf{e} \quad \overset{!}{\longrightarrow} \quad \min. \tag{4}$$

This is an equivalent formulation to finding $\hat{\mathbf{x}}$ with

$$||\mathbf{S} \cdot \hat{\mathbf{x}} - \mathbf{b}||_{\ell_2}^2 = \min_{\mathbf{x} \in \mathbb{R}^m} ||\mathbf{S} \cdot \mathbf{x} - \mathbf{b}||_{\ell_2}^2, \tag{5}$$

since the sum in equation (4) is precisely the squared ℓ_2 norm in equation (5). For a minimum of $E(\mathbf{x})$ the derivative of $E(\mathbf{x})$ with respect to $\mathbf{x}$ has to vanish

$$\frac{\partial E(\mathbf{x})}{\partial \mathbf{x}} \overset{!}{=} 0. \tag{6}$$

In matrix form $E(\mathbf{x})$ can be written as

$$\begin{aligned} E(\mathbf{x}) &= \mathbf{r}^T \mathbf{r} \\ &= (\mathbf{S} \cdot \mathbf{x} - \mathbf{b})^T (\mathbf{S} \cdot \mathbf{x} - \mathbf{b}) \\ &= (\mathbf{x}^T \mathbf{S}^T - \mathbf{b}^T)(\mathbf{S} \cdot \mathbf{x} - \mathbf{b}) \\ &= \mathbf{x}^T \mathbf{S}^T \mathbf{S} \mathbf{x} - \mathbf{b}^T \mathbf{S} \mathbf{x} - \mathbf{x}^T \mathbf{S}^T \mathbf{b} + \mathbf{b}^T \mathbf{b} \end{aligned}$$

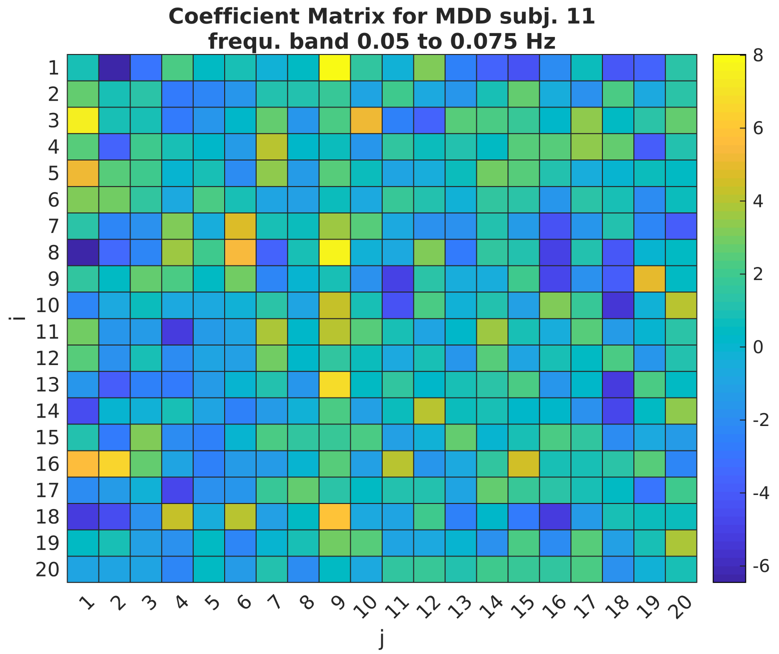

Figure 4: **Kuramoto Coefficient matrix $\mathbf{K}^{\text{res}}$ for subject** 11 **of group MD.** Time courses were filtered to the frequency band $0.05-0.075$Hz. The diagonal elements which correspond to coefficients $K_{i,i}$ are set to 1.

The derivative with respect to $\mathbf{x}$ is then

$$\begin{aligned}\frac{\partial E(\mathbf{x})}{\partial \mathbf{x}} &= 2\mathbf{S}^T\mathbf{S}\mathbf{x} - (\mathbf{b}^T\mathbf{S})^T - \mathbf{S}^T\mathbf{b} \\ &= 2\mathbf{S}^T\mathbf{S}\mathbf{x} - 2\mathbf{S}^T\mathbf{b}\end{aligned}$$

Plugging in this derivative into equation (6) and dividing by 2 yields

$$\mathbf{S}^T\mathbf{S}\hat{\mathbf{x}} \overset{!}{=} \mathbf{S}^T\mathbf{b}. \tag{7}$$

Thus, by solving the so called Gaussian normal equations (7) we obtain an approximated solution $\hat{\mathbf{x}}$ for the original problem (3), which is optimal in the sense of least squared errors.

2.5 Kuramoto coupling coefficients

The calculated Kuramoto coupling coefficients ($\mathbf{K}^{\text{res}}$, see Sec. 2.2 in the main paper) of one subject can be nicely visualized with a heatmap, where we can directly compare estimated coupling strengths between different IBNs. See Fig. 4 as an example for one subject.

3 Artificial data simulation

3.1 Solving the simulation model to generate phase courses

To obtain simulated phase courses, we have to solve the system of ODEs given in Appendix A, Equ. (15) of the main paper. As the numerical solver of the ODE system, we choose not to work with the same as when calculating the coefficients (i.e., Euler's method). This prevents to simply get out what we put in. The solver used for the simulations is the classical Runge-Kutta algorithm (Schwarz and Köckler, 2011) also called RK-4 with step size 1. One iterations step of the described simulation is given by the following formula

$$\varphi_i(s+1) = \varphi_i(s) + \frac{1}{6} \cdot (g_1 + 2 \cdot g_2 + 2 \cdot g_3 + g_4) + n \cdot \varepsilon_i(s). \tag{8}$$

We define a function g as

$$g(\theta, f, w, \mathbf{a}, \mathbf{l}, \boldsymbol{\varrho}) := f + \frac{w}{r} \cdot \sum_{j=1}^{r} a_i \cdot l_i \cdot \sin(\varrho_j - \theta) \tag{9}$$

with θ, f and w real numbers, $\mathbf{a}$, $\mathbf{l}$ and $\boldsymbol{\varrho} \in \mathbb{R}^r$. The terms g_1 to g_4 in formula (8) are then given by

$$\begin{aligned} g_1 &= g(\varphi_i(s), \omega_i, d, \mathbf{k}_i, \mathbf{m}_i, \boldsymbol{\varphi}(s)) \\ g_2 &= g(\varphi_i(s) + 0.5 \cdot g_1, \omega_i, d, \mathbf{k}_i, \mathbf{m}_i, \boldsymbol{\varphi}(s)) \\ g_3 &= g(\varphi_i(s) + 0.5 \cdot g_2, \omega_i, d, \mathbf{k}_i, \mathbf{m}_i, \boldsymbol{\varphi}(s)) \\ g_4 &= g(\varphi_i(s) + g_3, \omega_i, d, \mathbf{k}_i, \mathbf{m}_i, \boldsymbol{\varphi}(s)) \end{aligned}$$

Providing initial values $\varphi_i(0)$, $i = 1, \ldots, r$, formula (8) iteratively yields the phase courses. Note, that we switched from t to s as the function variables, since we are now considering iteration steps instead of time steps. $\mathbf{k}_i$ and $\mathbf{m}_i$ denote the $i-$th row of the matrices $\mathbf{K}$, $\mathbf{M}$, respectively and $\boldsymbol{\varphi}(s)$ is the vector of all r phase values at iteration step s.

3.2 Technical parameters

In this section, we provide more details about the chosen parameters of the artificial data simulation (see Appendix A of the main paper). The size of our data set is chosen as $s \times r \times T = 24 \times 20 \times 300$, which represents $s = 24$ instances (representing the subjects) with $r = 20$ phase courses (representing the IBN's activity phase courses) with $T = 300$ measure points, where each measure point corresponds to an activity value every two seconds. This is the same size as the exploratory data set and corresponds to a typical clinical study setting. We set the number of iteration steps in the RK-4 procedure to 3000 and afterwards cut off the first 300 values to eliminate possible transient phenomena. We then down sample by choosing every 9th value to get a time series of length 300 as for the empirical data.The initial phases for each region of each subject are randomly chosen within $[-\pi, \pi)$ and the eigenfrequencies are drawn from a Gaussian distribution $\mathcal{N}(0.0625, 0.0125)$. This corresponds to the frequency band with the statistically highly significant finding for the empirical data. The frequency band needs to be chosen rather narrow since this is a prerequisite of the Kuramoto model. For each of the 24 subjects the 20×20 coupling coefficients matrix $\mathbf{K}^{\text{o}}$ is randomly initialized with values ranging from approximately -20 to 20 as this is the range for most of the calculated Kuramoto coefficients derived with our method for data in the same frequency band. Each subject is randomly assigned a score value (representing the Ham-D) between 0 and 40 resulting in the vector $\mathbf{s}$. To maintain a certain balance between synchronization and de-synchronization, the weight factor d in equation (8) needed to be set such that the oscillators deviate from their respective eigenfrequencies, but at the same time the influence needs to be damped to prevent highly noisy and strongly oscillating phase courses. Starting above a d value of 0.6, coupling leads to severe impact on the phase time courses, to an extent that the overall spectral properties of the time courses are distorted. As this does not reflect a realistic scenario we limited our simulations to d values within the range of $[0.01, 0.6]$. Gaussian noise was added with intensity levels within the range $[0.5, 3]$ resulting in 5% to 30% noise, which falls into the regime of stochastic resonance in modelling of fMRI time courses (Deco et al., 2009).

Fig. 5 shows a typical example of a synthetically generated IBN phase course within freq3. The according procedure is described in Appendix A of the main paper.

3.3 Rediscovery analysis

We analyzed in how far the exact couplings parametrically depending on the score $\mathbf{s}$ could be recovered by the analysis pipeline. We, therefore, separated couplings into three categories being the couplings actually parametrically manipulated within the closed cluster in the ground truth data (amounting to 156 couplings, upper left part of the matrix in Fig. 6, couplings 'within the patterns' $\mathbf{N}_{\text{s}}$ and $\mathbf{N}_{\text{as}}$ respectively, termed IC), those couplings between an IBN from within the closed correlations pattern and an 'outside' IBN (amounting to 182 couplings, bridging couplings termed BC), and those only connecting IBNs from outside the set of manipulated coefficients (amounting to 42 couplings, lower right part of the matrix in Fig. 6, reference couplings termed RC). In this last group of reference couplings the detection of a significant parametric dependence of the $\mathbb{K}^{\text{res}}$

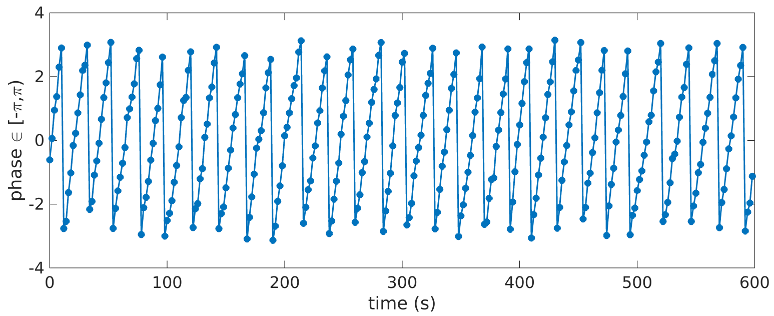

Figure 5: **Typical simulated phase course.** The figure shows an (already down sampled) phase course as typically produced by the simulation process described in Appendix A of the main paper. We used the convention to state phase values within the interval $[-\pi, \pi)$. Generated values (circles) are linearly interpolated for better visualization.

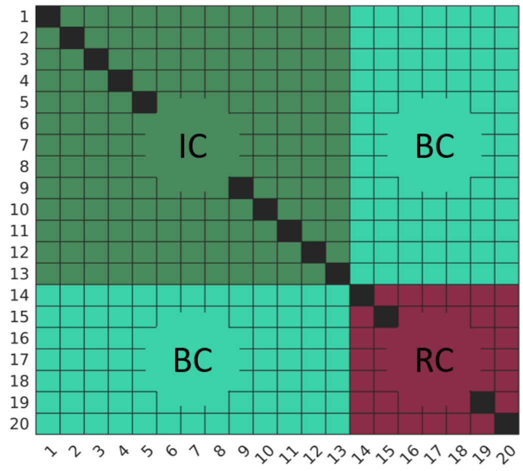

Figure 6: **Rediscovery analysis regions.** The total of 380 coefficients are divided into the three areas of 156 within the set of manipulated coefficients (IC, **dark green**), 182 bridging coefficients (BC, **turquoise**) and 42 reference coefficients outside the set of manipulated coefficients (RC, **dark red**). Diagonal elements are marked in black, since they are not calculated with our method and, therefore, are excluded in all the analysis.

parameters on the score s was regarded as false positive. In the group of 'bridging couplings' such a dependence would highlight at least one region actually affected by true parametric dependence on s. We evaluated the rediscovery of significant clusters with two versions.

Metric 1. The numbers of significant coefficients in the respective regions IC, BC and RC are divided by the total number of significant coefficients, i.e. within the significant set.

Metric 2. The numbers of significant coefficients in the respective regions IC, BC and RC are divided by the total number of significant coefficients, i.e. within the significant set, and it is additionally scaled by the size of the respective region (i.e., 156 for IC, 182 for BC and 42 for RC).

Note that the total number of coefficients in the cluster can vary largely across runs and parameter settings. Metric 1 expresses IC, BC and RC shares of the number of significant coefficients in the cluster and, therefore only accounts for the size of the cluster, but not the sizes of the IC, BC and RC areas. Metric 2 accounts for both. Since metric 1 does not account for the sizes, it gives some insight irrespective of the choice of the set of coefficients with induced correlations (i.e., manipulated coef-

ficients), which influences the rediscovery values for IC, BC and RC due to the scaling with their sizes.

We focused on scenarios for which the significance of retrieving a significant coupling set by the analysis pipeline was ≤ 0.05. We, therefore, evaluated the two metrics in two versions.

Version A. For each run, we calculate the respective IC, BC and RC metrics, for all (n, d) settings. For the respective run, we only keep the values of significant (≤ 0.05) settings. We then calculate the mean over all remaining metric values in all runs for IC, BC and RC, respectively.

Version B. For each run, we calculate the respective IC, BC and RC metrics, for all (n, d) settings. We then calculate the mean value over all runs for each (n, d) setting. From these mean matrices we only take into account the (n, d) combinations for which the median P-value over the six runs was ≤ 0.05. We then calculated the mean over the remaining metric values for IC, BC and RC, respectively.

4 Additional empirical data results

4.1 Dependence of K on head movement and cardiac rate

In the exploratory data set, patients with greater disease severity exhibited more motion during the scan ($P < 0.034$, Spearman correlation). When testing for a dependence of K on head movement, we also found a significant relation ('all': $P = 0.028$, 'pos': $P = 0.014$, 'neg': $P > 0.05$). To analyse the contributions of movement and Ham-D on the sets of Ks we followed the Baron and Kenny's steps for mediation (1986). The direct effect (not mediated by Ham-D) of movement on K was calculated by regressing Ham-D out of the movement parameters and using the resulting regressor in the set-level statistical analysis and vice versa. Results of the mediations are shown in Fig. 7. While Ham-D almost completely mediates the dependence of motion on K, motion does not mediate the dependence of Ham-D on K.

In the test data set a bite bar had been used. There was neither a dependence of K on head movement nor of Ham-D on head movement in the test patient data set.

There was no dependence of K on the cardiac rate in the exploratory or test data set.

4.2 Group difference in FC

Fig. 8 shows the set of couplings being at trend ($P_c = 0.063$) in the group contrast between MD patients and HC. Patients displayed higher values than controls (see Sec. 3.1.1 in the main paper).

5 Additional simulation results

5.1 Additional mask matrices

In the main paper in Appendix A we presented cluster permutation test results for the two correlation patterns $\mathbf{N}_{\text{s}}$ and $\mathbf{N}_{\text{as}}$ and three mask matrices. We performed simulations with three additional mask matrices. All six masks are depicted in Fig. 9. Fig. 10 and 11 show the collective results for all masks for $\mathbf{N}_{\text{s}}$ and $\mathbf{N}_{\text{as}}$, respectively.

5.2 Parameters determined by the analysis pipeline deviated from zero to a very limited extent

For all runs of all parameter settings we applied a Wilcoxon signed rank test for all coefficients $K_{i,j}^{\text{res}}$ (Bonferroni corrected for multiple testing with 380), if coefficients were significantly different from zero. There was no substantial evidence for the detected coefficients to significantly deviate from zero (see Appendix A of the main paper). Table 2 shows the collective Wilcoxon signed rank test results for the coefficients $\mathbb{K}^{\text{res}}$ for all correlation patterns and mask scenarios.

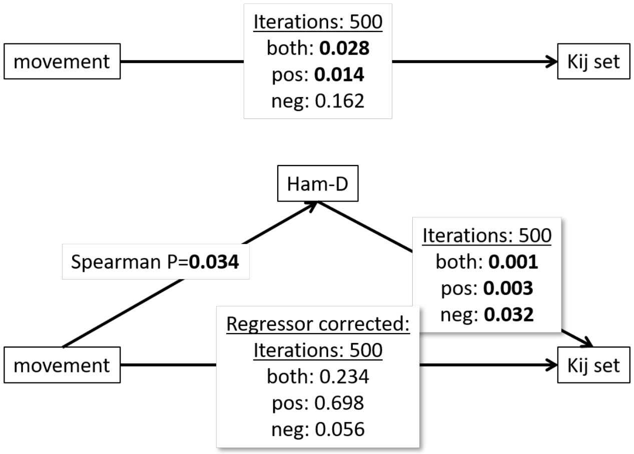

(a) Total mediation by Ham-D

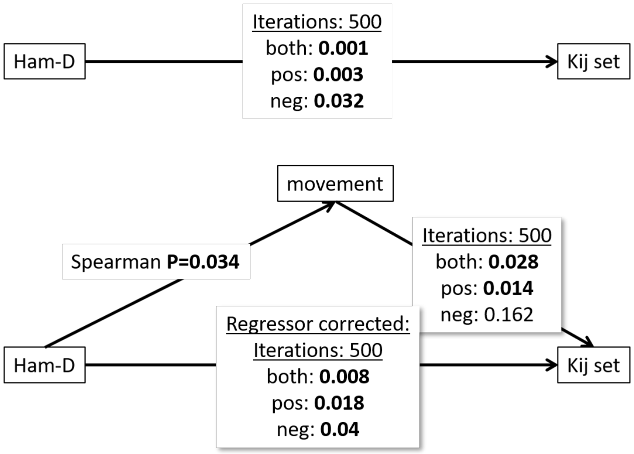

(b) No mediation by movement

Figure 7: Dependence of K on Ham-D and movement

5.3 Results for random initial matrices

We further present in Fig. 12 the median P-value results for the random initial matrices $\mathbb{K}^o$ (see Appendix A in the main paper).

5.4 Recovered sets of couplings are shifted to joining regions

For each mask and correlation pattern we calculated the two metrics (do not account for area size vs account for size) for IC, BC and RC, respectively and evaluated both versions A and B (consider all significant (n, d) settings across runs vs consider only settings which are significant in the median of runs). Table 3 summarizes the results. For metric 1 we can see for both versions, that for all scenarios $BC > IC > RC$ with three exceptions, where $IC > BC > RC$. When accounting for the sizes of the areas, the ratio of false positives increases. However, IC and BC combined still contain the most significant coefficients. Furthermore, also the SD increases disproportionately for RC compared to IC and BC, which makes the rations for recovered false positives less stable.

We conclude, the reliability of significant coefficients can be mainly addressed on an IBN level, but not necessarily for the specific coefficient. In general, couplings of group BC had the highest likelihood of being included into a significant set.

In Table 3 all results for both metrics and both evaluation versions are presented for the all correlation pattern and mask settings.

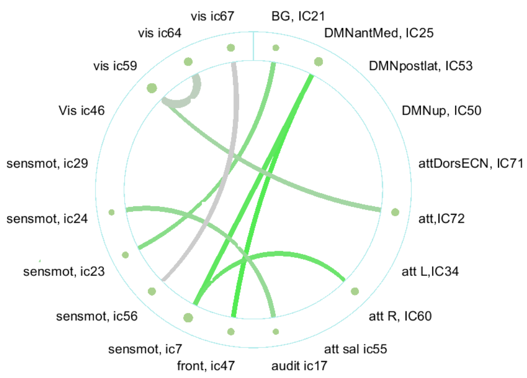

Figure 8: **Set of increased FC couplings in MD versus HC.** Line width scales with median difference. Dots mark the IBNs with names according to Allen et al., 2011 and scale in size with overall median difference towards all other IBNs.

	run 1	run 2	run 3	run 4	run 5	run 6
$\mathbf{N}_\text{s}$ $\mathbf{M}_{1/1}$	3	1	4	0	2	1
$\mathbf{N}_\text{s}$ $\mathbf{M}_{0.5/0}$	1	6	1	1	1	1
$\mathbf{N}_\text{s}$ $\mathbf{M}_{1/0}$	2	1	1	0	1	1
$\mathbf{N}_\text{s}$ $\mathbf{M}_{1/0.5}$	3	3	0	0	3	3
$\mathbf{N}_\text{s}$ $\mathbf{M}_{1/0.25}$	2	2	2	1	3	3
$\mathbf{N}_\text{s}$ $\mathbf{M}_{1/0.1}$	2	2	3	2	1	2
$\mathbf{N}_\text{as}$ $\mathbf{M}_{1/1}$	0	1	3	0	1	1
$\mathbf{N}_\text{as}$ $\mathbf{M}_{0.5/0}$	0	4	1	0	0	1
$\mathbf{N}_\text{as}$ $\mathbf{M}_{1/0}$	2	0	0	1	3	2
$\mathbf{N}_\text{as}$ $\mathbf{M}_{1/0.5}$	2	0	2	1	1	1
$\mathbf{N}_\text{as}$ $\mathbf{M}_{1/0.25}$	2	1	1	4	0	0
$\mathbf{N}_\text{as}$ $\mathbf{M}_{1/0.1}$	1	0	1	0	1	1

Table 2: Table values and caption have been adjusted to the new results of the synthetic experiments evaluated with the new model. **Wilcoxon signed rank test results for $\mathbb{K}^{\textbf{res}}$ of artificial data.** For all the matrices, masks, runs and (n, d) combinations at most one coefficient resulted in a positive test. The table summarizes how often that occurred. As an example: The number 3 in the top left spot means, that for correlation pattern $\mathbf{N}_\text{s}$ and mask $\mathbf{M}_{1/1}$, there were 3 different (n, d) combinations in run 1, for which one of the 380 coefficients yielded a positive Wilcoxon signed rank test (Bonferroni corrected with 380). It only occurred twice that for a single (n, d) combination more than one coefficient resulted in a positive test. This was only the case in run 2 for the setting $\mathbf{N}_\text{s}$ with $\mathbf{M}_{0.5/0}$ and in run 5 for the setting $\mathbf{N}_\text{as}$ with $\mathbf{M}_{1/0}$, for which in one (n, d) combination, 3, respectively 2, coefficients were seen as significantly different from 0 by the test. The cluster permutation test in these cases yielded a significant result, however, the coefficients with a positive signed rank test were all not part of this significant set.

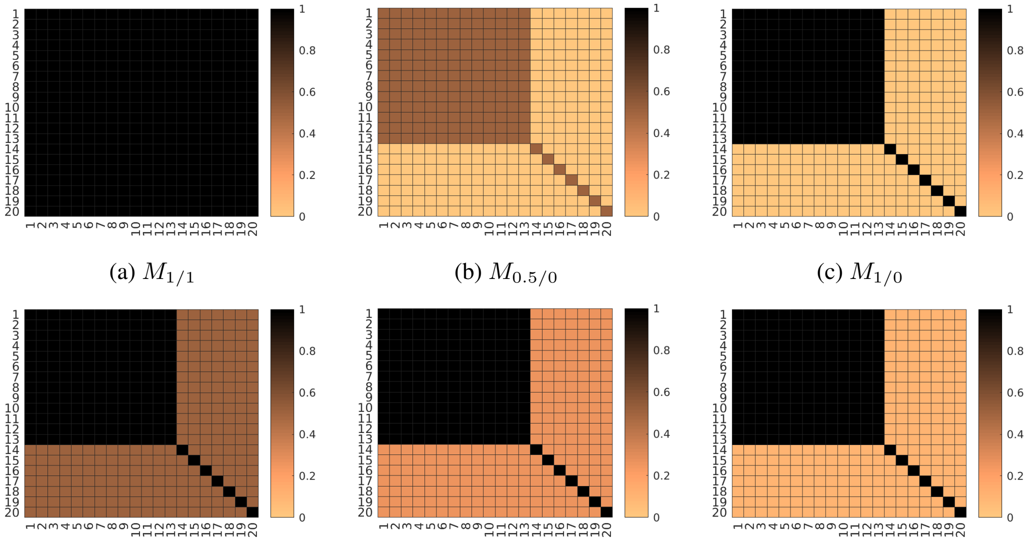

(a) $M_{1/1}$ (b) $M_{0.5/0}$ (c) $M_{1/0}$

(d) $M_{1/0.5}$ (e) $M_{1/0.25}$ (f) $M_{1/0.1}$

Figure 9: **Mask matrices.** Each subfigure shows one of the six 20×20 mask matrices used in the experiments. The sub captions contain their naming, where the subscripts in $\mathbf{M}_{\text{in/out}}$ should be interpreted as follows: The number at the position 'in' is the weight for the coefficients with distinct correlations ('inside the correlation structures' $\mathbf{N}_s$ and $\mathbf{N}_{as}$ respectively), while the number in the subscript position 'out' is the weight for all coefficients with a very weak correlation ('outside these correlation structures'). For example, in the case of (d) $M_{1/0.5}$, the coefficients with induced strong positive or negative correlations are twice as active as the remaining coefficients.

		metric 1		metric 2*				metric 1		metric 2*	
$\mathbf{N}_s$		version A	version B	version A	version B	$\mathbf{N}_{as}$		version A	version B	version A	version B
$\mathbf{M}_{1/1}$	IC	0.48 ± 0.13	Nan	0.30 ± 0.08	Nan	$\mathbf{M}_{1/1}$	IC	0.39 ± 0.17	Nan	0.25 ± 0.11	Nan
	BC	0.44 ± 0.11	Nan	0.24 ± 0.06	Nan		BC	0.50 ± 0.17	Nan	0.28 ± 0.09	Nan
	RC	0.08 ± 0.06	Nan	0.20 ± 0.14	Nan		RC	0.11 ± 0.09	Nan	0.27 ± 0.21	Nan
$\mathbf{M}_{0.5/0}$	IC	0.38 ± 0.16	0.34 ± 0.08	0.25 ± 0.11	0.22 ± 0.05	$\mathbf{M}_{0.5/0}$	IC	0.27 ± 0.16	0.25 ± 0.08	0.17 ± 0.10	0.16 ± 0.05
	BC	0.51 ± 0.15	0.54 ± 0.05	0.28 ± 0.08	0.30 ± 0.03		BC	0.60 ± 0.16	0.60 ± 0.08	0.33 ± 0.09	0.33 ± 0.05
	RC	0.11 ± 0.06	0.12 ± 0.05	0.26 ± 0.14	0.29 ± 0.12		RC	0.14 ± 0.10	0.15 ± 0.07	0.32 ± 0.24	0.36 ± 0.17
$\mathbf{M}_{1/0}$	IC	0.34 ± 0.19	0.23 ± 0.12	0.22 ± 0.12	0.15 ± 0.08	$\mathbf{M}_{1/0}$	IC	0.27 ± 0.15	0.27 ± 0.08	0.17 ± 0.10	0.17 ± 0.05
	BC	0.52 ± 0.17	0.58 ± 0.11	0.28 ± 0.09	0.32 ± 0.06		BC	0.59 ± 0.17	0.59 ± 0.10	0.32 ± 0.09	0.32 ± 0.05
	RC	0.15 ± 0.12	0.19 ± 0.08	0.35 ± 0.28	0.46 ± 0.18		RC	0.14 ± 0.09	0.14 ± 0.05	0.33 ± 0.21	0.33 ± 0.13
$\mathbf{M}_{1/0.5}$	IC	0.47 ± 0.14	0.47 ± 0.03	0.30 ± 0.09	0.31 ± 0.02	$\mathbf{M}_{1/0.5}$	IC	0.41 ± 0.21	0.37 ± 0.00	0.26 ± 0.13	0.24 ± 0.00
	BC	0.44 ± 0.13	0.44 ± 0.02	0.24 ± 0.07	0.24 ± 0.01		BC	0.49 ± 0.20	0.50 ± 0.00	0.27 ± 0.11	0.28 ± 0.00
	RC	0.08 ± 0.05	0.08 ± 0.01	0.19 ± 0.11	0.19 ± 0.03		RC	0.10 ± 0.10	0.12 ± 0.00	0.25 ± 0.25	0.29 ± 0.00
$\mathbf{M}_{1/0.25}$	IC	0.45 ± 0.16	Nan	0.29 ± 0.11	Nan	$\mathbf{M}_{1/0.25}$	IC	0.39 ± 0.20	0.36 ± 0.10	0.25 ± 0.13	0.23 ± 0.06
	BC	0.46 ± 0.15	Nan	0.25 ± 0.08	Nan		BC	0.51 ± 0.18	0.52 ± 0.11	0.28 ± 0.10	0.28 ± 0.06
	RC	0.09 ± 0.07	Nan	0.22 ± 0.17	Nan		RC	0.10 ± 0.09	0.12 ± 0.09	0.23 ± 0.22	0.30 ± 0.20
$\mathbf{M}_{1/0.1}$	IC	0.42 ± 0.18	0.28 ± 0.05	0.27 ± 0.12	0.18 ± 0.03	$\mathbf{M}_{1/0.1}$	IC	0.35 ± 0.18	0.31 ± 0.08	0.23 ± 0.12	0.20 ± 0.05
	BC	0.47 ± 0.15	0.57 ± 0.03	0.26 ± 0.08	0.31 ± 0.02		BC	0.55 ± 0.18	0.58 ± 0.08	0.30 ± 0.10	0.32 ± 0.04
	RC	0.11 ± 0.09	0.15 ± 0.06	0.26 ± 0.21	0.36 ± 0.15		RC	0.10 ± 0.09	0.11 ± 0.05	0.24 ± 0.21	0.26 ± 0.12

Table 3: **Retrieved cluster coefficients.** For each correlation pattern and mask matrix combination, the mean values and standard deviations for the two metrics (1 and 2) and two evaluation versions (A and B) are given. We can observe for metric 1 where we do not consider the size of the areas IC, BC and RC, that the BC area had, in general, the highest likelihood of containing significant cluster coefficients. For most cases, false positives (i.e., RC) had the lowest likelihood. In specific cases, e.g. $\mathbf{N}_s$ and $\mathbf{M}_{1/0.5}$, the IC had the highest likelihood. $*$Values of metric 2 are naturally very small since they are not only divided by the total number of coefficients with significant correlations, but additionally by the size of the respective area. For better readability and comparability these values were multiplied by 100. Here, we can observe, that the proportion of false positives increases in comparison to the other areas, especially as significance (low P-values) in the median is more prominent (see Fig. 10 and 11). However, SD also increases disproportionately for RC. Further, IC and BC combined remain the largest portion of recovered coefficients.

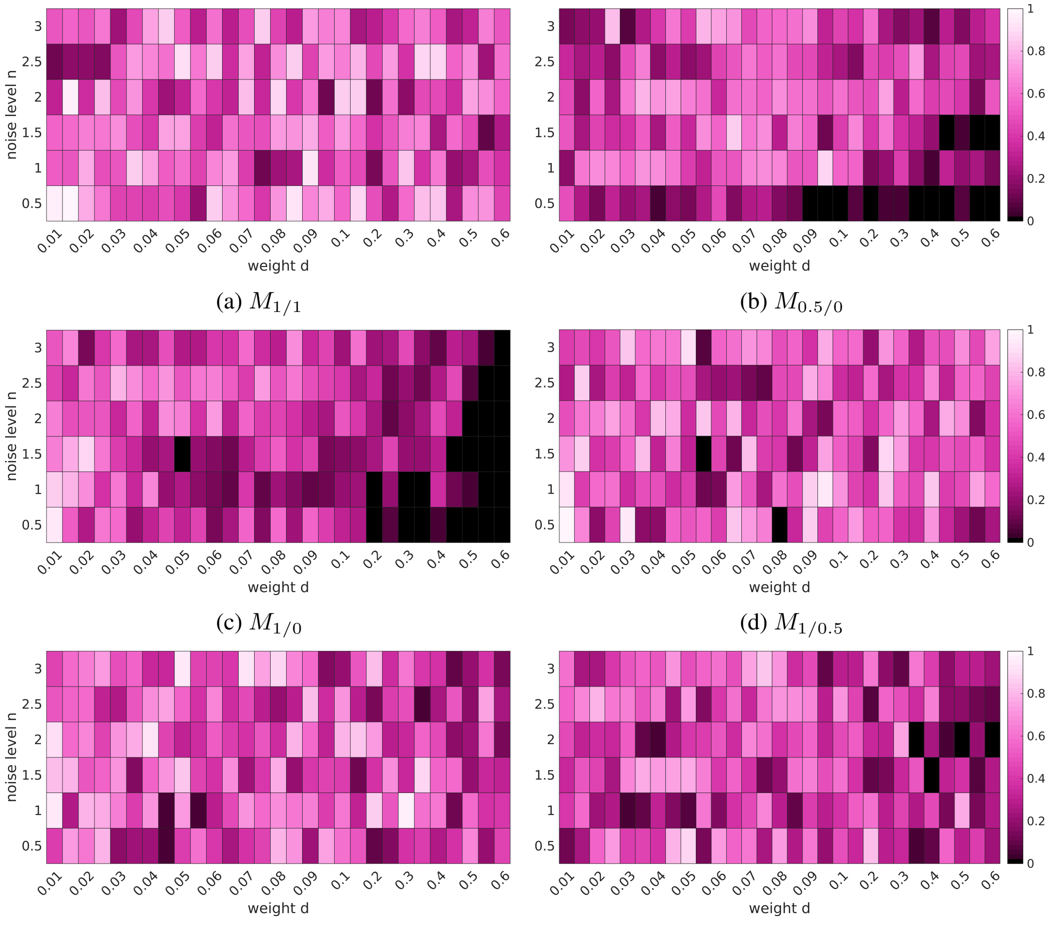

(a) $M_{1/1}$ (b) $M_{0.5/0}$

(c) $M_{1/0}$ (d) $M_{1/0.5}$

(e) $M_{1/0.25}$ (f) $M_{1/0.1}$

Figure 10: **Results for $\mathbf{N_s}$.** The above figures show the median P-values over the 6 runs for all (n, d) combinations, $\mathbb{K}^c$ with the correlation pattern $\mathbf{N}_s$. Each subplot shows the results for one of the 6 mask matrices. Cells with significant P-values are colored in black. It shows that increasing d leads to a better capacity of the analysis pipeline to retrieve the significant dependence on the sore $\mathbf{s}$ from the artificial data, when the contrast in dependence of the $\mathbb{K}^c$ coefficients outside the set of coefficients with strong correlations are set to 0 ((b) and (c)) or have very weak influence (f). When the contrast in dependence of the $\mathbb{K}^c$ coefficients outside the set of coefficients with strong correlations to within this set is weak to moderate ((d) and (e)), or even non present (a) significance is only retrieved randomly. Added noise ε in the coupling process has a detrimental effect on recovering the dependence on $\mathbf{s}$.

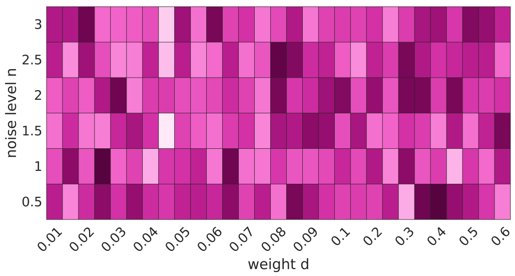

(a) $M_{1/1}$

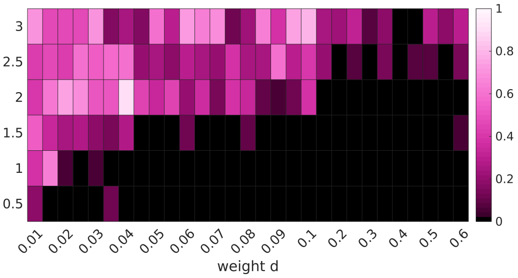

(b) $M_{0.5/0}$

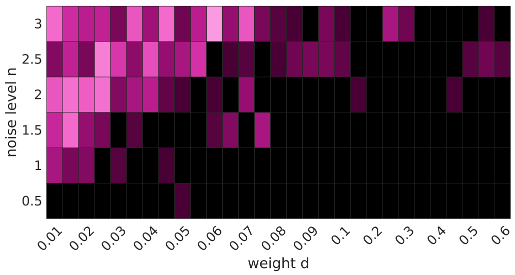

(c) $M_{1/0}$

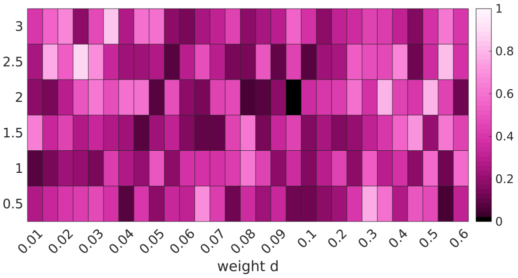

(d) $M_{1/0.5}$

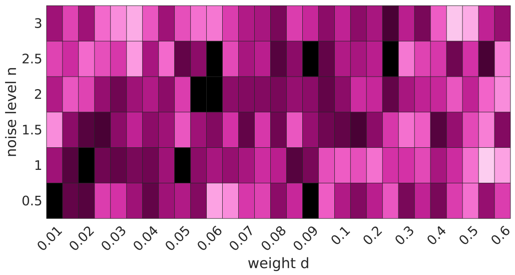

(e) $M_{1/0.25}$

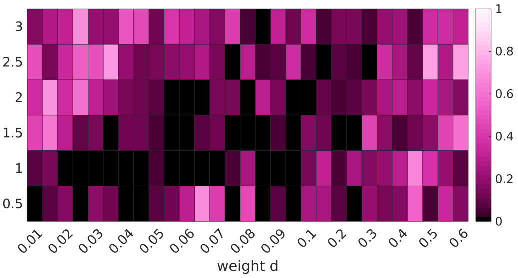

(f) $M_{1/0.1}$

Figure 11: **Results for $\mathbf{N}_{\mathbf{as}}$**. The above figures show the median P-values over the 6 runs for all (n, d) combinations, $\mathbb{K}^c$ with the correlation pattern $\mathbf{N}_{\text{as}}$. Each subplot shows the results for one of the 6 mask matrices. Cells with significant P-values are colored in black. It shows that increasing d leads to a better capacity of the analysis pipeline to retrieve the significant dependence on the sore $\mathbf{s}$ from the artificial data, when the contrast in dependence of the $\mathbb{K}^c$ outside the set of coefficients with induced strong correlations are set to 0 ((b) and (c)) or have very weak (f) to moderate (e) influence. When the contrast in dependence of the $\mathbb{K}^c$ coefficients outside the set of coefficients with strong correlations to within this set is weak (d) or non present (a) significance is only retrieved randomly. Added noise ε in the coupling process has a detrimental effect on recovering the dependence on $\mathbf{s}$. Significant effects can be retrieved more prominently for this correlation pattern than for the symmetric correlation pattern $\mathbf{N}_{\text{s}}$ (see Fig. 10).

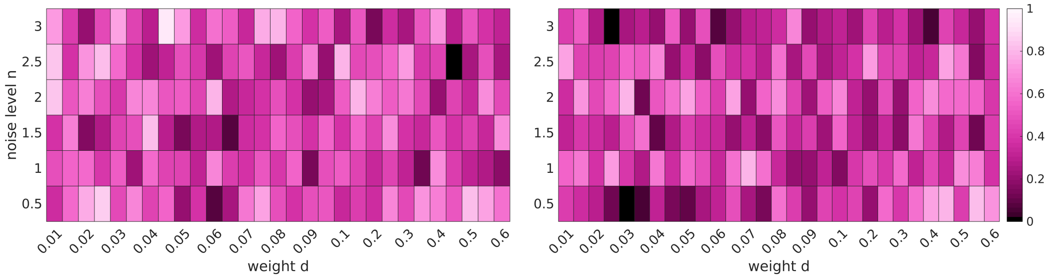

(a) Results for $\mathbb{K}^{\mathrm{o}}$ and $\mathbf{M}_{1/1}$. The median P-values show no dependence on (n, d). significant settings only appear randomly.

(b) Results for $\mathbb{K}^{\mathrm{o}}$ and $\mathbf{M}_{1/0}$. The median P-values show no dependence on (n, d) even if a large portion of coefficients is excluded in the simulation process.

Figure 12: **Results on random initial matrices.** Cells with significant P-values are colored in black. **(a)** If we simulate phase courses where the coefficients do not show a significant correlation with the the score $\mathbf{s}$ (i.e., $\mathbb{K}^{\mathrm{o}}$ and mask $\mathbf{M}_{1/1}$), the significance in the statistical analysis shows a random behavior. Some parameter combinations indicate the finding of a network cluster associated with $\mathbf{s}$, but this does not correspond to the intensity of the coefficients in the simulation or the noise level. In general, fewer and less significant results are found. **(b)** To determine, if the results are associated with the correlation structure rather than with the applied masks $\mathbf{M}_{1/0}$ or $\mathbf{M}_{0.5/0}$, we repeat the experiment for the original random correlated matrices $\mathbb{K}^{\mathrm{o}}$, but set the same coefficients to zero as in the cases of $\mathbf{N}_{\mathrm{s}}$ and $\mathbf{N}_{\mathrm{as}}$. Again the results show a random behavior, which indicates a strong impact from the correlations and a weaker impact of the exclusion of certain coefficients by setting them to zero on the results.

References

Acebrón, J. A., Bonilla, L. L., Pérez Vicente, C. J., Ritort, F., & Spigler, R. (2005). The kuramoto model: A simple paradigm for synchronization phenomena. *Rev. Mod. Phys.*, *77*, 137–185. https://doi.org/10.1103/RevModPhys.77.137

Allen, E. A., Erhardt, E. B., Damaraju, E., Gruner, W., Segall, J. M., Silva, R. F., Havlicek, M., Rachakonda, S., Fries, J., Kalyanam, R., Michael, A. M., Caprihan, A., Turner, J. A., Eichele, T., Adelsheim, S., Bryan, A. D., Bustillo, J., Clark, V. P., Feldstein Ewing, S. W., … Calhoun, V. D. (2011). A Baseline for the Multivariate Comparison of Resting-State Networks. *Frontiers in Systems Neuroscience*, *5*, 1–23. https://doi.org/10.3389/fnsys.2011.00002

Arenas, A., Díaz-Guilera, A., Kurths, J., Moreno, Y., & Zhou, C. (2008). Synchronization in complex networks. *Physics Reports*, *469*(3), 93–153. https://doi.org/10.1016/j.physrep.2008.09.002

Bocharova, M. (2014). Modelling and analysis of amplitude, phase and synchrony in human brain activity patternsa. *Dissertation at the University College London, Centre for Mathematics and Physics in the Life Sciences and Experimental Biology (CoMPLEX).*

Deco, G., Jirs, V., McIntosh, A. R., Sporns, O., & Kötter, R. (2009). Key role of coupling, delay, and noise in resting brain fluctuations. *Proceedings of the National Academy of Sciences of the United States of America*, *106*, 10302–10307. https://doi.org/10.1073/pnas.0901831106

Deco, G., Tagliazucchi, E., Laufs, H., Sanjuán, A., & Morten, L. (2017). Novel intrinsic ignition method measuring local- global integration characterizes wakefulness and deep sleep. *eNeuro*, *4*, 1–12.

Epperson, J. F. (2013). *An introduction to numerical methods and analysis*. John Wiley; Sons.

Kuramoto, Y. (1975). Self-entrainment of a population of coupled non-linear oscillators. In H. Araki (Ed.), *International symposium on mathematical problems in theoretical physics* (pp. 420–422). Springer Berlin Heidelberg.

Schwarz, H. R., & Köckler, N. (2011). *Numerische mathematik* (Vol. 6). Vieweg+Teubner Verlag. https://doi.org/10.1007/978-3-8348-8166-3

Winfree, A. T. (1967). Biological rhythms and the behavior of populations of coupled oscillators. *Journal of Theoretical Biology*, *16*(1), 15–42. https://doi.org/https://doi.org/10.1016/0022-5193(67)90051-3
